# Scalable Total Synthesis of (+)-Desmethylxestospongin B

**DOI:** 10.1021/acs.joc.4c00779

**Published:** 2024-05-29

**Authors:** Alana K. Borum, Karen Y. Chen, Armen Zakarian

**Affiliations:** Department of Chemistry and Biochemistry, University of California, Santa Barbara, California 93106, United States

## Abstract

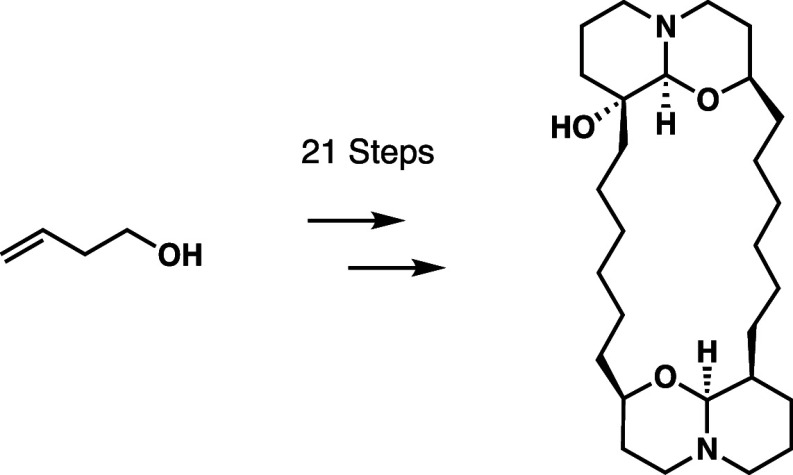

Herein, the execution of synthetic strategies solving scalability issues observed in the original route is reported, increasing the total yield by 50% compared to the previously disclosed synthesis. A notable restructuring of the route’s initial steps to reach a common allylic alcohol intermediate employs a highly stereoselective epoxidation method and avoids superfluous protecting group manipulations while limiting dependence on kinetic resolution in establishing stereochemistry for four of the six chiral centers in (+)-desmethylxestospongin B. Different protecting group strategies to avoid problems with their subsequent removal were considered and enacted; to this end, material was retained as byproducts were suppressed. While the lactam semireduction under Birch conditions requires further investigation, the updated synthesis of (+)-desmethylxestospongin B reported here made it more scalable, affording 0.37 g of this natural product for continued biological studies.

## Introduction

(+)-Desmethylxestospongin B (dmXe B) is a marine natural product, holding attention for its structural complexity and bioactivity. Xestospongin and araguspongine compounds were isolated from species *Xestospongia exigua* and structurally identified to be composed of two bis-1-oxaquinolizidine heterocycles bound by two equivalent hexamethylene chains.^1−3^ Variations in the structure of these compounds include *C*_2_ symmetry or lack thereof as well as the presence or absence of C9 and/or C9′ hydroxylation. Some of these xestospongin compounds—notably, dmXe B—were observed to affect mitochondrial respiration by blocking inositol triphosphate receptors in the endoplasmic reticulum, leading to cellular autophagy and allowing selective tumor cell apoptosis. This phenomenon has been observed in treating MDA-MB-231 cell lines with increasing concentrations of dmXe B against normal MCF10A cells.^4,5^ While the promise of targeted cancer treatments remains attractive, the natural source of dmXe B has been depleted while biological studies are still ongoing. Our lab has developed and reported a 22-step total synthesis for this compound in 2021.^5^

**Scheme 1:**
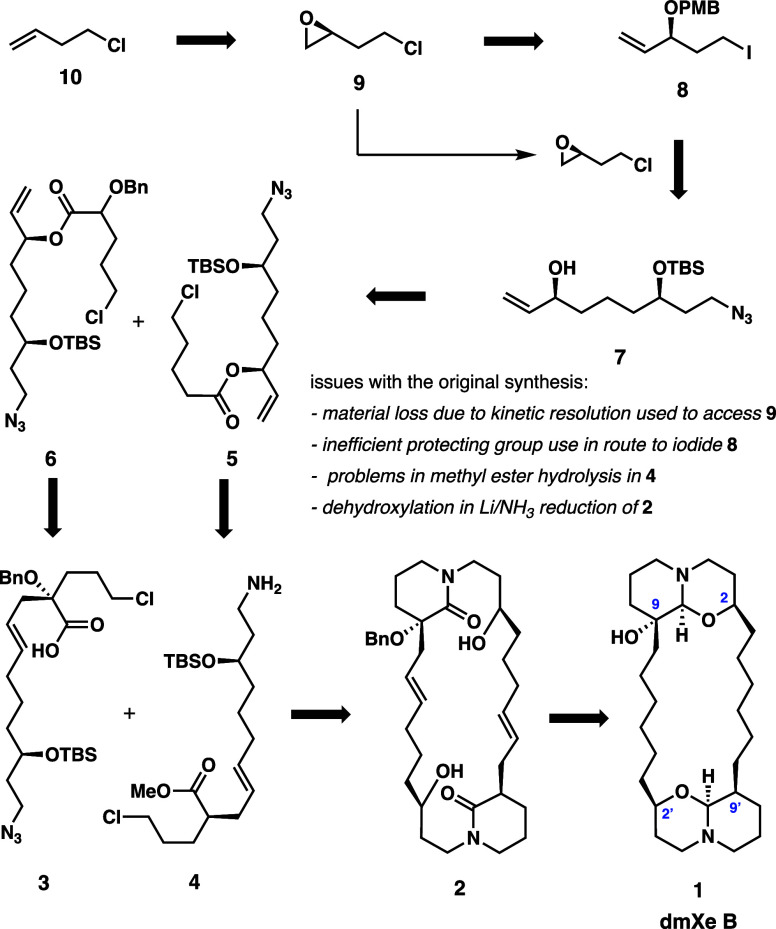
Original Synthetic Path to dmXe B

The syntheses of other xestospongin compounds were executed by other groups. Hoye et al.’s synthesis^6^ of (+)-xestospongin A and Baldwin et al.’s synthesis^7^ of (+)-araguspongine B and (−)-xestospongin A both approach C2-symmetrical targets lacking C9/9′ hydroxylation through dimerization strategies, which has proven highly effective in the production of these targets. In Baldwin et al.’s case, the syntheses of enantiomers of the naturally produced xestospongins were crucial in the assignment of absolute stereochemistry. An original route was required in the synthesis of an asymmetric, C9/9′ hydroxylated xestospongin (as is dmXe B), so it could be produced from simple starting materials.^5^ The strategy enacted previously in our group (shown in Scheme 1) involves ring closure of the bis-1-oxaquinolizidine heterocycles immediately following partial reduction of δ-lactam in **2** to a hemiaminal intermediate and then hydrogenation to give the saturated hexamethylene fragments. In functionalizing fragments **3** and **4** independently, the desired macrolactam is assembled in a stepwise manner, leaving no room for homodimerization in the formation of a bis-lactam intermediate, followed by ring closure of the primary amide intermediate to δ-lactams in **2**, occurring upon exposure to LiN(SiMe_3_)_2_. In the case of acid **3** and amine **4**, the established stereochemistry and functionalization at C9 and C9′ is established by separate Ireland-Claisen rearrangement reactions, their conditions determined by the α-substitution of the corresponding allylic ester substrates **5** and **6**. In the case of **4**, the rearrangement is followed by esterification and azide reduction to amine to allow for the stepwise formation of the two crucial amide bonds in **2**. These individual allylic esters **5** and **6** are derived from esterification of azido alcohol **7** with two different acid fragments, and **7** itself is synthesized through a coupling reaction between chiral epoxide **9** and alkyl iodide **8** by the use of a higher-order cuprate. Intermediate **8** itself is given by methylenation and alcohol protection of chiral epoxide **9**, which is furnished through an epoxidation of **10** with MCPBA followed by kinetic resolution.^5^

While the original synthesis remains effective in achieving a nonsymmetric functionalization present in dmXe B, a few outstanding questions remained following the initial completion of the route. Solving these problems would substantially improve the route, its efficiency, and its scalability and ultimately provide larger amounts of this biologically remarkable target compound. Roughly half the material in making chiral epoxide **9** (which is used twice in the synthesis to establish stereochemistry for four centers at C2, C2′, C9, and C9′ on the two oxaquinolizidine heterocycles) had to be discarded after kinetic resolution with Jacobsen’s catalyst. Reducing dependence on this material could be achieved with an asymmetric transformation to establish at least two of the four aforementioned stereocenters. The first 12 steps of the synthesis showed some material inefficiency, furthered by the superfluous alcohol protection to access alkyl iodide **8**, as the *para*-methoxybenzyl (PMB) group would need to be removed a mere four steps after its introduction in a 22-step synthesis. Introduction of the PMB group was difficult as conversion would not reach completion and the substrate had to be resubmitted to the reaction; a new approach that could avoid the requirement of this protection/deprotection sequence would certainly improve the productivity of these initial steps. On a separate note, methyl ester hydrolysis in **4** to access the intermediate for macrolactamization was accompanied by removal of the silyl-protecting groups. This desilylated byproduct was observed upon the rapidly applied acidic workup conditions required in order to avoid the formation of the six-membered lactone byproduct, which could form under the basic conditions of the hydrolysis. Finally, the Birch reduction of the δ-lactam units showed a 54% yield of the desired product, as well as an 11% yield of the α-dehydroxylation to the araguspongine B (Ar B) precursor. The reaction requires further improvement to suppress this α-dehydroxylation.

## Results and Discussion

**Scheme 2:**
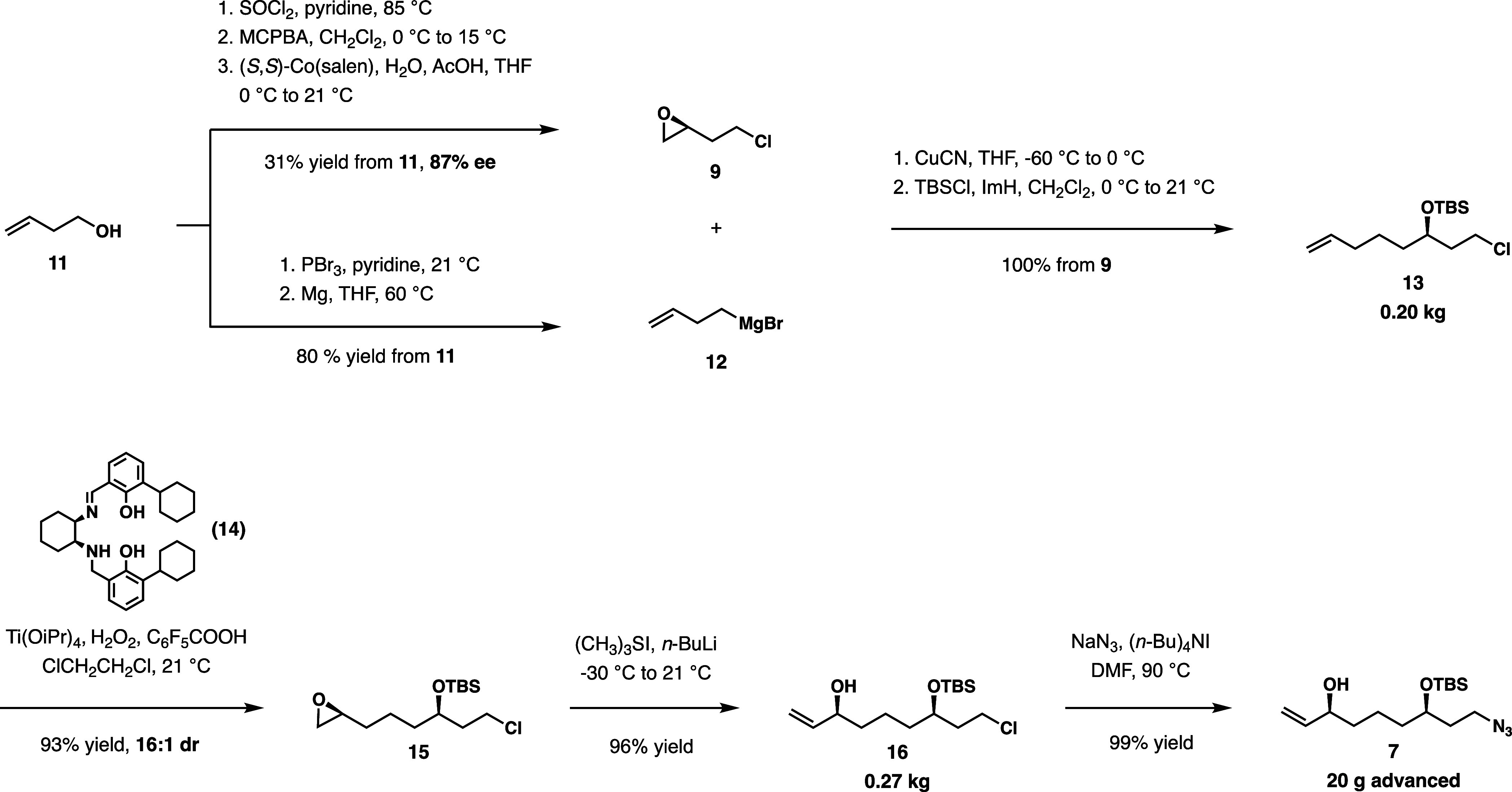
Improved Synthesis of Key Azido Alcohol **7**

To answer these outstanding questions regarding the scalability of this important target, several modifications were proposed and executed in an updated synthesis. The original route was significantly adjusted in the first 12 steps to improve efficiency. To this end, an novel asymmetric epoxidation method is applied to establish the stereocenter in terminal epoxide **15** and subsequently allylic alcohol **16** (Scheme 2), which is to be translated to C9 and C9′ stereochemistry and functionalization; this further reduces dependence on the production of epoxide **9**. In this manner, the inessential and cumbersome PMB protection to give **8** is no longer required. To prevent early-stage desilylation with the acidic workup conditions applied after methyl ester hydrolysis, this challenging intermediate is replaced instead with an allyl ester, as the mild conditions for its cleavage would not affect the silyl ether groups. Finally, in a hypothesis to reduce α-dehydroxylation in the Birch reduction, the benzyl ether, which gives the C9 hydroxyl group, is changed from Bn to PMB so the ether is first cleaved selectively, with DDQ and not concurrently, to the reduction step. Having the free hydroxyl group at C9 was hypothesized to suppress loss of this functional group as the rate of α-dehydroxylation was expected to be slower than α-debenzyloxylation.

The modified synthesis begins with 3,4-buten-1-ol (**11**), which is distributed to prepare 4-bromo-1,2-butene (which will be converted to Grignard reagent **12**) as well as (*S*)-2-(2-chloroethyl)oxirane (**9**), as depicted in Scheme 2. The chiral epoxide is generated by the same sequence as in the original synthesis. Chlorodehydroxylation of the substrate with thionyl chloride, followed by epoxidation with MCPBA, and then kinetic resolution using Jacobsen’s catalyst to provide the chiral epoxide **9** in 31% yield over three steps and 87% ee. Once Grignard reagent **12** is prepared from 4-bromo-1,2-butene, it is immediately submitted to reaction with **9** using a catalytic amount of copper(I) cyanide.^8^ In the adjustment to include the copper-catalyzed Grignard addition to the chiral epoxide in the synthesis, the reaction yielded pure material in its crude state, allowing purification to be postponed until after the subsequent silylation of the resulting alcohol. Furthermore, the reaction remained robust without any byproducts observed even with increasing concentration from 0.18 to 0.5 M, quantitatively processing reactions up to 17 g with reduced amounts of solvent, reliably providing the alcohol product in quantitative yields. Scales and concentration exceeding 17 g and 0.5 M, respectively, can still be explored. After the product alcohol is silylated, the crude product is purified through a silica plug, giving 100% yield of **13** over these two steps.

**Scheme 3:**
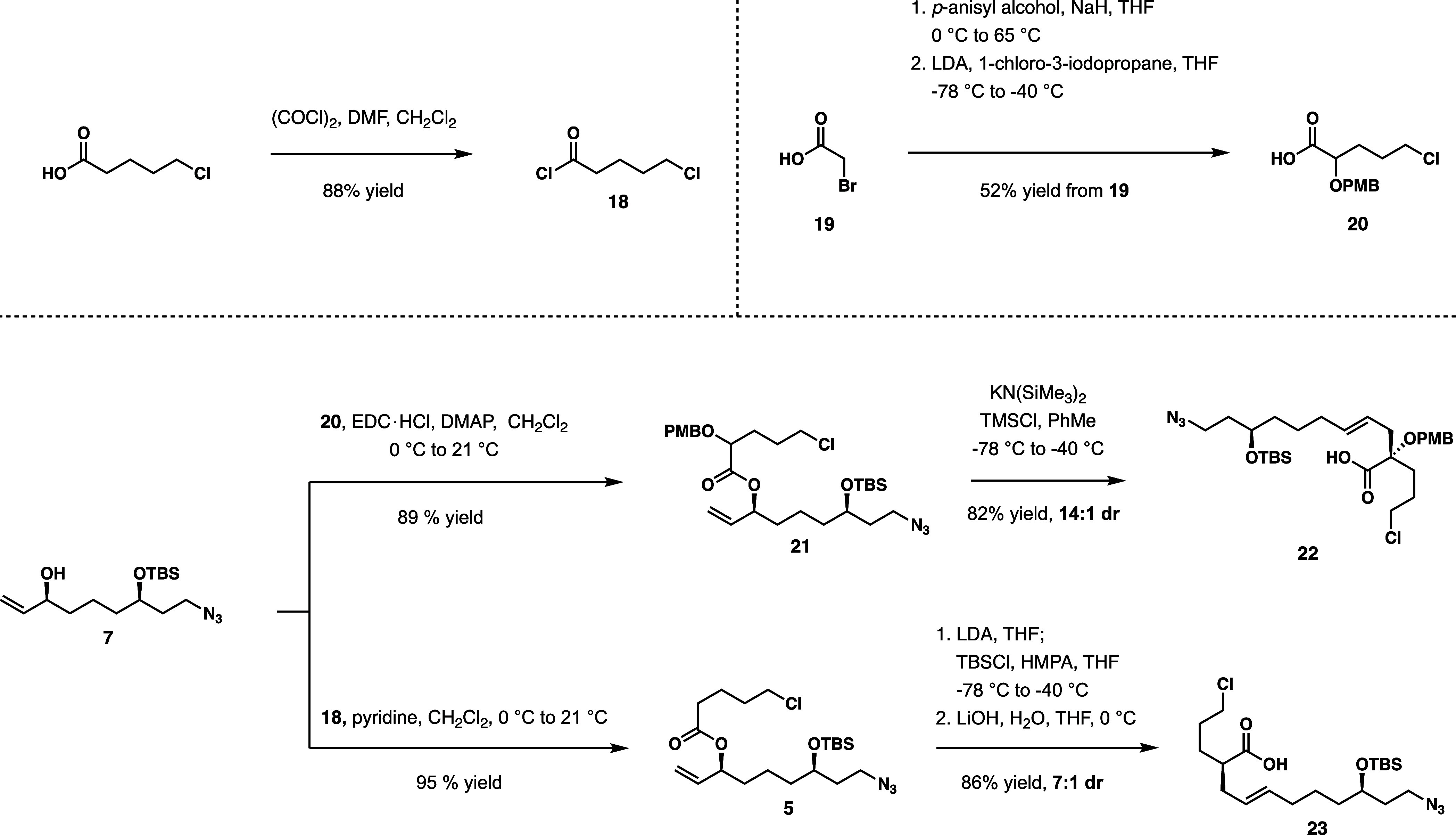
Synthesis of Allylic Esters and Subsequent Ireland-Claisen Rearrangements

Following the synthesis of olefin **13**, asymmetric epoxidation is employed to increase material efficiency in establishing stereochemistry for two out of four centers set by the kinetic resolution used in the initial synthesis of dmXe B. While numerous and reliable methods to perform asymmetric epoxidations exist, there were few options to allow the transformation to take place on terminal alkenes such as **13** or its alcohol precursor. Initial attempts at remote stereocontrol with desilylated **13** using conditions such as VO(acac)_2_/*t*-BuOOH proved unproductive, with minimal conversion observed. Attention was turned toward Berkessel et al.’s unique work, in which asymmetric epoxidations could be carried out on simple terminal alkenes through the in situ preparation of a catalyst formed from titanium isopropoxide and salalen ligand **14**.^9^ In preparation for incorporating this work into the synthetic route, it was decided to perform this asymmetric epoxidation on **13** instead of its alcohol precursor, as the epoxide generated from the unprotected alcohol was observed to undergo intramolecular etherification to form a tetrahydropyran byproduct when purified on silica gel. Material up to 140 g was submitted through this asymmetric epoxidation to give a 93% yield. Not only that but also the newly employed asymmetric epoxidation method afforded the product with 15.7:1 dr, compared with the 24:1 dr observed in the original route, effectively matching stereocontrol from the original route with a more straightforward, high-yielding method. Methylenation of the epoxide by use of an ylide prepared from trimethylsulfonium iodide and *n*-butyllithium, which is followed by azide substitution of the chloride, gave allylic alcohol **7**. In modifying the front end of the synthesis, the yield of this key common intermediate **7** was increased from 14 to 31% from **10** as compared to the original route and the number of steps was reduced by two. Having successfully constructed intermediate **7**, this compound is then distributed to synthesize the two acid fragments **22** and **23** (Scheme 3). Compound **5** was prepared through the esterification of **7** with 5-chloropentanoyl chloride. Ester **21** was constructed through the synthesis of carboxylic acid **20**, prepared by substitution of 1-bromoacetic acid with *p*-methoxybenzyl alcohol, and then direct alkylation with 1-chloro-2-iodopropane, which is then submitted to esterification with intermediate **7**, eventually providing the hydroxylation at C9 of dmXe B.

Allylic esters **21** and **5** are then submitted to Ireland-Claisen rearrangement conditions. Ester **5** is submitted to this rearrangement under standard conditions using lithium diisopropylamine. Through this rearrangement, preference for the (*E*)-enolate gives predictable stereochemistry favoring the desired diastereomer **23** with a 7:1 dr.

With **21**, the Ireland-Claisen rearrangement was accomplished with KN(SiMe_3_)_2_ in toluene, which provided optimal conditions for the corresponding transition on the α-substituted allylic ester substrate. Previous work demonstrated that the rearrangement of α-alkoxy-substituted allylic esters under such conditions gives preference for the (*Z*)-enolate, resulting from potassium chelation to the silyl ketene acetal intermediate,^10^ affording the product acid **22** at an excellent 14:1 dr.

**Scheme 4:**
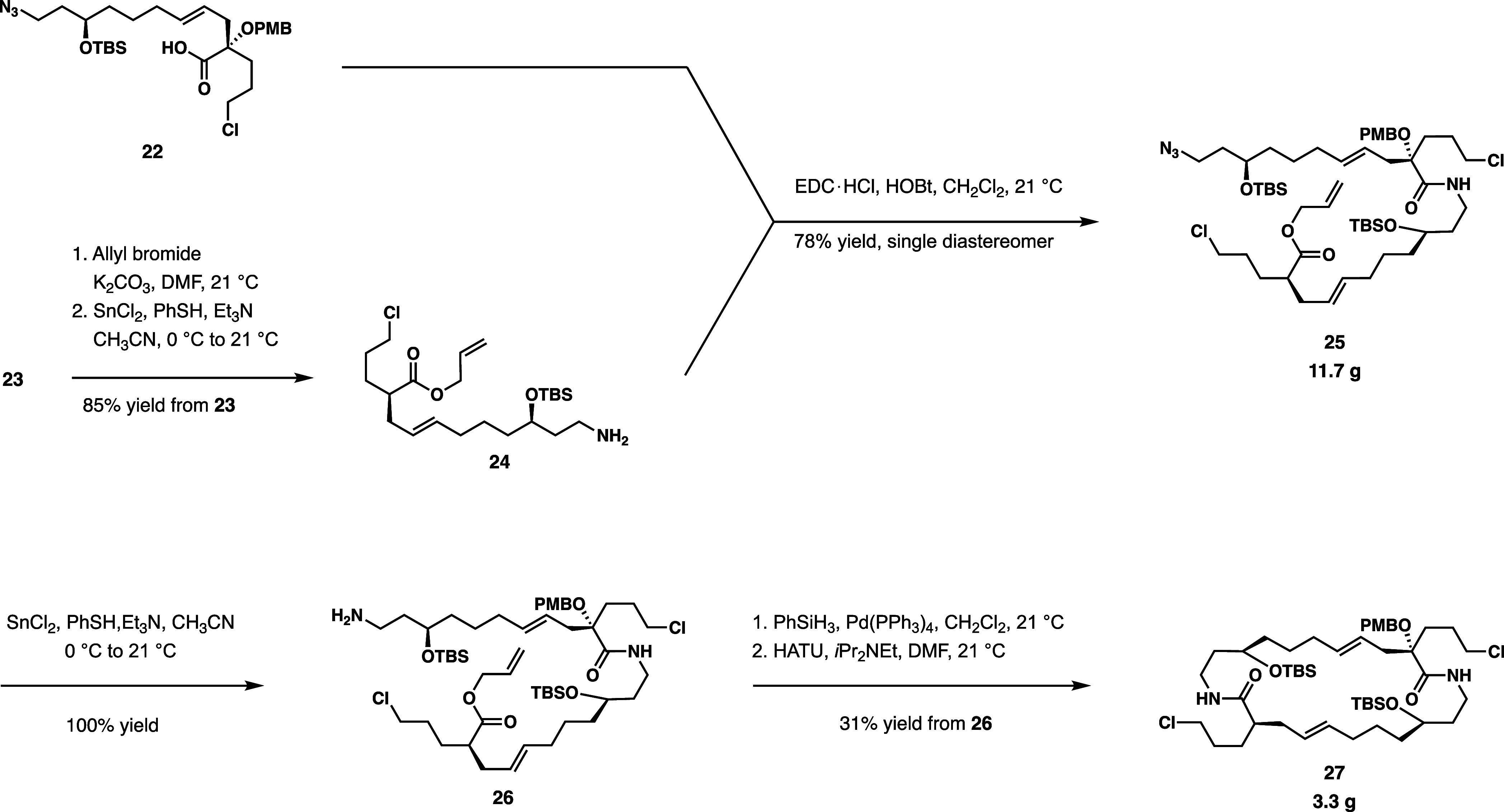
Fragment Convergence; Macrolactamization

Acid **23** undergoes allylation, followed by azide reduction to provide amine **24** (Scheme 4). It should be noted that the conditions for the azide reduction were slightly adjusted from what was previously reported^5^ in reducing the presence of thiophenol from 6 to 4.5 equiv, as reported in literature.^11,12^ This modification circumvented the previously observed thiophenol substitution of chloride in **24**. Furthermore, the concentration of the reaction was increased from 0.1 M (to the substrate) to 1.0 M, in order to prevent the overconcentration of crude material given by the previous procedure, which required acetonitrile removal *in vacuo* before aqueous workup could quench the reducing agent. At higher reaction concentrations, the solution could be diluted in methylene chloride and submitted to aqueous workup immediately. With these procedural adjustments in place, the initial temperature for the addition of azide substrate was lowered from 21 to 0 °C to counteract the exothermic reaction at the increased concentration. In this rendition of the azide reduction with tin(II) chloride, quantitative yields were achieved without any unwanted byproducts observed from the previous procedure.

With carboxylic acid **22** and amine **24** in hand, amide coupling to advanced intermediate **25** is performed. Following the purification of amide **25**, the azide is reduced using the same tin(II) chloride conditions, as previously employed in constructing **24**. The acid is liberated cleanly with the removal of the allyl group using phenylsilane and palladium tetrakis(triphenylphosphine). This method avoided drastic changes in pH, as was the case with the methyl ester intermediate of the original route. Then, the macrolactamization takes place under high dilution in 31% yield to enable the formation of a 24-membered ring in bis(lactam) **27**.

**Scheme 5:**
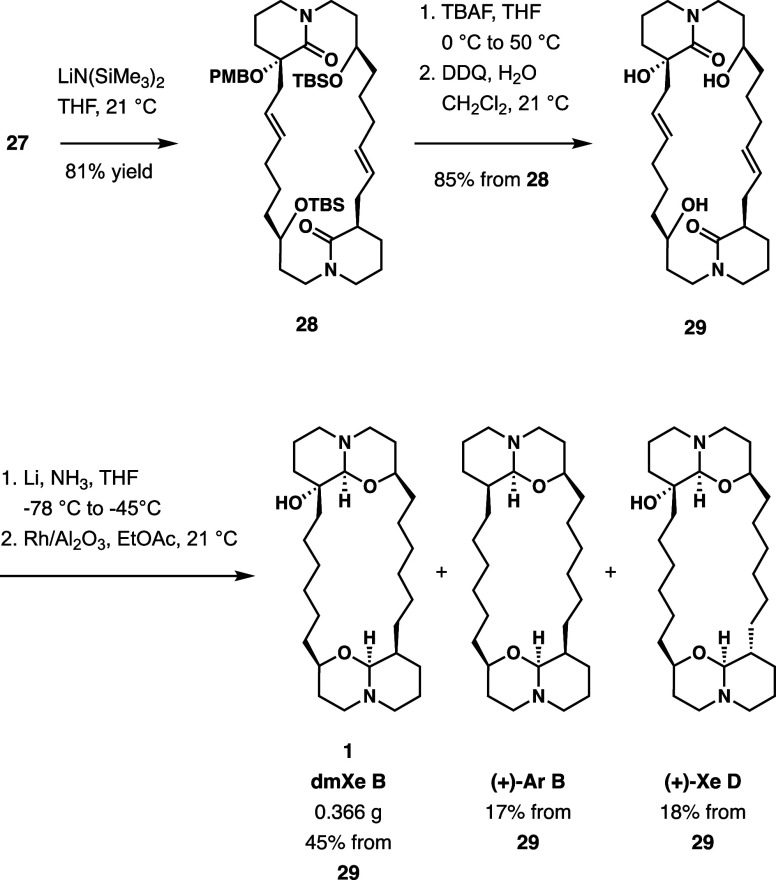
Synthesis of dmXe B

The first six-membered rings comprising the two functionalized bis-oxaquinolizidine heterocycles are formed in an intramolecular *N*-substitution of the amide with the chloride, mediated by LiN(SiMe_3_)_2_. This reaction demanded great care as epimerization at the C9′ position will take place under the strongly basic environment. Incremental addition of LiN(SiMe_3_)_2_ in THF to the substrate solution was therefore required, titrating the reaction to completion. Thus, epimerization was minimized with the minor amount of byproduct readily removed by column chromatography, providing **28** as a single diastereomer (Scheme 5).

**Scheme 6:**
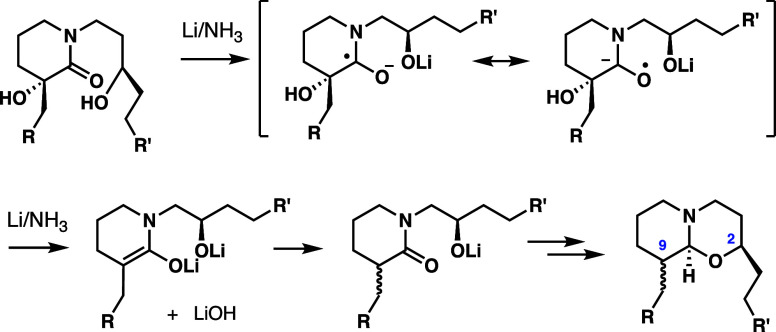
Proposed Pathway for α-Deoxygenation

Following this ring closure, desilylation of **28** is accomplished in this stage in preparation for the final construction of the bis-oxaquinolizidine heterocycles. After removal of the PMB group (85% yield over two steps), compound **29** is subjected to Birch reduction conditions. Careful direction was required for this reaction, as simply following that of the original synthesis led to an incomplete reaction with only one of the bis-oxaquinolizidine rings formed. In efforts to drive the reaction to completion, the reaction required extension of the reaction time (30 min instead of 15 min) at a higher temperature (−45 °C instead of −78 °C). Upon completion of this step, α-dehydroxylation was unfortunately observed (Scheme 6), as well as epimerization, giving precursors to (+)-araguspongine B and (+)-xestospongin D, respectively, with the majority of the material corresponding to the precursor to dmXe B. These results were likely due to the extended reaction time required for the Birch reduction. Following the final submission of the mixture to hydrogenation with rhodium on alumina, the mixture was separated into its pure components by using reverse-phase column chromatography, affording 0.37 g (45% yield over two steps) of dmXe B.

## Conclusions

In summary, a successful synthesis of dmXe B was completed, with several modifications enhancing scalability issues, enabling resumption of biological studies on this natural product. With these adjustments in place, the yield of the common intermediate **7** (following the modified beginning of the synthesis) was increased more than twofold, decreasing dependence on chiral material obtained by kinetic resolution. The use of an allyl ester increased the total yield by avoiding premature desilylation prior to macrolactamization. However, α-dehydroxylation at C9 was observed at a higher ratio with the free hydroxyl group at C9 compared to the benzyl ether in the same position from the previous synthesis, which indicates that further studies are required to improve the Birch reduction step of this synthesis. Nevertheless, the completion of this project saw achievement of the primary goal, as the overall yield of dmXe B (from **10**) improved by about 50% from 2.2% to 3.3% in 20 steps.^14^

## Experimental Section

### General Information

All reactions were carried out under an inert atmosphere of dry argon in oven- or flame-dried glassware unless the reaction procedure states otherwise. Tetrahydrofuran (THF) and diethyl ether (Et_2_O) were distilled from sodium-benzophenone in a continuous still under an atmosphere of argon. Toluene was distilled over sodium under an inert atmosphere of argon. Dichloromethane, diisopropylamine, pyridine, triethylamine, and chorotrimethylsilane were distilled from calcium hydride in a continuous still under an atmosphere of argon. Diisopropylethylamine (Hünig’s base) was distilled from calcium hydride under an inert atmosphere of dry argon and stored over calcium hydride. Reaction temperatures were controlled by IKA ETS-D4 fuzzy thermo couples. Room temperature reactions were carried out between 20 and 22 °C. Analytical thin-layer chromatography (TLC) was performed using precoated TLC plates with Silica Gel 60 F 254 (EMD no. 5715-7) and visualized using combinations of UV, anisaldehyde, cerium ammonium molybdate, and potassium permanganate staining. Flash column chromatography was performed using 40–63 μm silica gel (Merck, Geduran, no. 11567-1) as the stationary phase. Proton nuclear magnetic resonance spectra were recorded at 400, 500, and 600 MHz on Varian Unity Inova and Bruker AVANCE NEO spectrometers. Carbon nuclear magnetic resonance spectra were recorded at 100, 125, and 150 MHz on Varian Unity Inova spectrometers and Bruker AVANCE NEO spectrometers. All chemical shifts were reported in δ units relative to those of tetramethylsilane. High-resolution mass spectral data were obtained by the Materials Research Laboratory at the University of California, Santa Barbara.

**Note:***n*-Butyllithium was purchased from Sigma-Aldrich (2.5 M solution in hexanes, catalog no. 230707–100 mL) and used directly as received. The reagent was titrated before use.

#### 4-Chloro-1,2-butene (**10**)

To freshly distilled 3,4-buten-1-ol (0.214 kg, 3.0 mol), freshly distilled pyridine (17 mL, 0.21 mol, 0.07 equiv) was added. The mixture was cooled to 0 °C before thionyl chloride (0.217 kg, 3.0 mol, 1.0 equiv) was added dropwise. After 0.5 h, the mixture was warmed to 21 °C and assembled a reflux condenser, distillation head, and collection flask over the reaction flask, and then the temperature was increased to 85 °C using an oil bath. ^1^H NMR monitoring indicated complete conversion after 2 h. The collected product was combined with the crude material, and distillation was performed at 95 °C. The distilled product was washed with a saturated NaHCO_3_ aqueous solution and dried with Na_2_SO_4_ to give 4-chloro-1,2-butene (0.231 kg, 2.6 mol, 86% yield) as a clear, colorless oil. ^1^H and ^13^C NMR spectral data matched those reported in the literature.^13^

#### 2-(2-Chloroethyl)oxirane

To a solution of **10** (0.225 kg, 2.5 mol) in 0.60 L of CH_2_Cl_2_ cooled to 0 °C, 75% MCPBA in H_2_O (0.628 kg, 2.7 mol, 1.1 equiv) was added in six portions. The temperature was allowed to reach but not exceed 15 °C. The reaction was monitored by ^1^H NMR; complete conversion was observed after 12 h. The reaction mixture was filtered and then placed in a −20 °C freezer overnight, filtered again, and cooled to 0 °C; then, Me_2_S (0.237 L, 3.2 mol, 1.3 equiv) was added and the temperature was increased to 21 °C. The presence of peroxides was checked by combining a 0.2 mL solution with 0.2 mL of AcOH and 20 mg of KI, confirming that peroxides were quenched. Removed CH_2_Cl_2_ was removed by distillation at 65 °C under atmospheric pressure (heating via an oil bath), and then distilled epoxide product was distilled under vacuum (35 mmHg) at 85 °C with the collection flask in a −78 °C cold bath (vapor temperature observed between 68 and 70 °C), providing 2-(2-chloroethyl)oxirane (0.214 kg, 81% yield) as a clear, colorless oil. ^1^H and ^13^C NMR spectral data matched those reported in the literature.^5^

#### (*S*)-2-(2-Chloroethyl)oxirane (**9**)

Freshly distilled 2-(2-chloroethyl)oxirane (59.7 g, 0.56 mol) was added to (*S*,*S*)-(salen)Co(II) (1.69 g, 2.80 mmol, 0.005 equiv) under an inert atmosphere, followed by 5.6 mL of THF and then acetic acid (0.32 mL, 5.6 mmol, 0.01 equiv). The mixture was cooled to 0 °C; then, DI H_2_O (5.6 mL, 0.31 mol, 0.55 equiv) was added dropwise to the reaction. The temperature was maintained for 20 min before warming to 21 °C. Monitoring by ^1^H NMR showed 52% conversion to diol after 96 h. All volatiles were collected by vacuum distillation at 0.2 mmHg at 70 °C (using an oil bath, increasing the temperature up to 95 °C to collect diol) in a vessel cooled to −78 °C. Next, they were dried over Na_2_SO_4_ (rinsing with CH_2_Cl_2_), and then rinses were combined with the crude product mixture. Distilled under atmospheric pressure to remove CH_2_Cl_2_ and THF, the remaining crude product mixture was then distilled under vacuum (35 mmHg) at 85 °C to isolate (*S*)-2-(2-chloroethyl)oxirane (26.4 g, 0.25 mol, 44% yield) into a flask cooled to −78 °C (vapor temperature observed between 50 and 52 °C). ^1^H and ^13^C NMR spectral data matched those reported in the literature.^5^

#### (*S*)-5-Chloropent-1-en-3-ol

A solution of Me_3_SI (67.2 mg. 0.33 mmol, 3.5 equiv) in 0.20 mL of THF was cooled to −30 °C before 2.1 M *n*-BuLi (0.15 mL, 0.32 mmol, 3.4 equiv) was added dropwise. After 0.5 h, the temperature was increased to −10 °C and (*S*)-2-(2-chloroethyl)oxirane (9.4 mg, 0.088 mmol) was added in solution with 0.25 mL of THF. The reaction was allowed to warm to ambient temperature over the course of 1 h, and then conditions were maintained for 1 h. When the reaction was complete by thin-layer chromatography, the sample was cooled to 0 °C and added to 1 mL of saturated NH_4_Cl_(aq)_ solution, followed by washing with 5 mL of brine and extraction with 5 mL of Et_2_O, drying with MgSO_4_, filtration, and then concentration in vacuo (with the bath set to 0 °C to prevent product evaporation). The crude product was submitted to the next reaction in its crude state.

#### (*S*)-1-(((5-Chloropent-1-en-3-yl)oxy)methyl)-4-methoxybenzene

To a solution of *p*-anisaldehyde (16 μL, 0.13 mmol, 1.5 equiv) in 0.60 mL of CH_2_Cl_2_, BF_3_·OEt (17 μL, 0.13 mmol, 1.5 equiv) was added dropwise at −40 °C and then the crude substrate (*S*)-5-chloropent-1-en-3-ol (0.088 mmol) in solution with 0.24 mL of CH_2_Cl_2_ was added dropwise to the reaction, followed by Et_3_SiH (50 μL, 0.31 mmol, 3.5 equiv). The reaction was warmed to −10 °C; these conditions were maintained for 1 h before quenching with 1 mL of DI H_2_O (the reaction did not reach completion by TLC analysis). The reaction was washed with 2 mL of saturated NaHCO_3(aq)_ solution and extracted with 5 mL of CH_2_Cl_2_. Next, it was dried with Na_2_SO_4_, filtered, and then concentrated in vacuo. The crude product was then purified by column chromatography (3% EtOAc in hexanes) to obtain (*S*)-1-(((5-chloropent-1-en-3-yl)oxy)methyl)-4-methoxybenzene (2.2 mg, 0.0091 mmol, 10% yield) as a pale-yellow oil. ^1^H and ^13^C NMR spectral data matched those reported in the literature.^5^ Er [93.5:6.5] (Chiralcel OD-H; 1% i-PrOH in hexanes; flow rate = 1.0 mL/min; detection at (230) nm; t1 = 5.18 min (major); t2 = 5.49 min (minor).

#### 4-Bromobut-1-ene

To freshly distilled 3,4-buten-1-ol (28.5 g, 0.40 mol) was added freshly distilled pyridine (9.0 mL, 0.11 mol, 0.28 equiv) at an ambient temperature, and then the mixture was cooled to 0 °C. Phosphorus tribromide (14.9 mL, 0.16 mol, 0.4 equiv) was added dropwise to the reaction, pausing addition if the reaction fumed excessively. The conditions were maintained for 0.5 h after the addition, and then the reaction was warmed to 21 °C. ^1^H NMR monitoring showed complete conversion after 2 h. The product was distilled under atmospheric pressure by heating to 150 °C (vapor temperatures 130–132 °C). The distilled product was washed with 40 mL of saturated NaHCO_3_ aqueous solution and dried with Na_2_SO_4_ (without rinsing the drying agent) to obtain 4-bromobut-1-ene (42.9 g, 0.32 mol, 80% yield) as a clear, colorless oil. ^1^H and ^13^C NMR spectral data matched those reported in the literature.^14^

#### But-3-en-1-ylmagnesium Bromide (**12**)

To a suspension of Mg turnings (5.47 g, 0.23 mol) and 10 μL of 1,2-dibromoethane in THF, 4-bromobut-1-ene (31.98 g, 0.24 mol, 1.05 equiv) was added dropwise at an ambient temperature. 0.5 h after substrate addition, the reaction was heated to 75 °C so reflux was observed. Conditions were maintained for 1 h until Mg turnings were completely consumed in solution. The solution was utilized immediately after it was cooled to ambient temperature for the following reaction.

#### (*R*)-1-Chlorooct-7-en-3-ol

To a solution of **9** (16.8 g, 0.16 mol) in 90 mL of THF, CuCN (0.678 g, 7.9 mmol, 0.05 equiv) was added. The mixture was cooled to −60 °C before 1.0 M but-3-en-1-ylmagnesium bromide solution in THF (0.225 L, 0.23 mol, 1.4 equiv) was added dropwise to the reaction. The conditions were maintained for 0.5 h after Grignard reagent addition, and then the reaction was allowed to warm to −30 °C over the course of 0.75 h. The temperature conditions were maintained for 0.5 h before allowing the reaction to warm to 0 °C over the course of 0.5 h. The reaction was kept at 0 °C for 0.75 h when TLC analysis indicated a complete reaction. 0.1 L of saturated NH_4_Cl_(aq)_ solution was added to quench at 0 °C and then warmed to 21 °C. The temperature conditions were maintained for 1 h before washing with brine and extracting with EtOAc. The solution was dried with Na_2_SO_4_, filtered, and concentrated in vacuo. This afforded the crude product (25.9 g), which was submitted to the next reaction without further purification.

#### (*R*)-*tert*-Butyl((1-chlorooct-7-en-3-yl)oxy)dimethylsilane (**13**)

The crude product (*R*)-1-chlorooct-7-en-3-ol (25.9 g, ∼0.16 mol) from the previous reaction was dissolved in 50 mL of CH_2_Cl_2_; imidazole (32.6 g, 0.48 mol, 3.0 equiv) was added in one portion. The reaction was cooled to 0 °C, and then *tert*-butyldimethylsilyl chloride (31.5 g, 0.21 mol, 1.5 equiv) was added in three portions. The temperature conditions were maintained for 0.5 h before warming to 21 °C. The reaction was stirred at an ambient temperature for 12 h; quenched with 50 mL of DI H_2_O; washed with DI H_2_O (2 × 30 mL) and then brine (30 mL) while extracting with 50 mL of hexanes; dried with Na_2_SO_4_; filtered; and then concentrated in vacuo. The crude material was purified through the use of a silica plug (hexanes) to afford **13** (43.3 g, 0.16 mol, 100% yield over two steps) as a clear, colorless oil. ^1^H NMR (600 MHz, CDCl_3_) δ 5.79 (ddt, *J* = 16.9, 10.2, 6.7 Hz, 1H), 5.01 (dq, *J* = 17.1, 1.7 Hz, 1H), 4.96 (ddt, *J* = 10.2, 2.3, 1.3 Hz, 1H), 3.87 (p, *J* = 5.7 Hz, 1H), 3.65–3.55 (m, 2H), 2.08–2.01 (m, 2H), 1.91–1.83 (m, 2H), 1.54–1.37 (m, 4H), 0.89 (s, 9H), 0.07 (d, *J* = 6.2 Hz, 6H). ^13^C{^1^H} NMR (500 MHz, CDCl_3_) 138.8, 114.8, 69.2, 42.1, 39.8, 36.8, 33.9, 26.0, 25.9, 24.3, 18.2, −4.2, −4.5. HRMS (ESI) *m*/*z*: [M + H] calcd for C_14_H_30_ClOSi 277.1754; found 277.1759.

#### *tert*-Butyl(((*R*)-1-chloro-6-((*S*)-oxiran-2-yl)hexan-3-yl)oxy)dimethylsilane (**15**)

The salalen ligand **14** was prepared as described according to literature.^8^ To a solution of **14** (12.3 g, 25 mmol, 0.05 equiv) in 0.230 L of CH_2_Cl_2_, titanium isopropoxide (7.5 mL, 25 mmol, 0.05 equiv) was added. The color was observed to change slightly from bright yellow/green to dark yellow/gold. After 2 h, CH_2_Cl_2_ was removed using vacuum distillation under 35 mmHg at 21 °C (ambient temperature water bath), providing the catalyst as a gold-colored solid.

To the flask containing the catalyst, substrate **13** (0.139 kg, 0.50 mol) was added, followed by 84 mL of 1,2-dichloroethane and pentafluorobenzoic acid (5.33 g, 25 mmol, 0.05 equiv). The color changed from dark yellow to red/orange after the addition of the acid additive. Next, 30% H_2_O_2(aq)_ (0.103 L, 1.0 mol, 2.0 equiv) was added. The reaction was allowed to stir at ambient temperature for 24 h before 30% H_2_O_2(aq)_ (0.103 L, 1.0 mol, 2.0 equiv) was added, and then additional 30% H_2_O_2(aq)_ (0.103 L, 1.0 mol, 2.0 equiv) was added again at 48 h. At 72 h, ^1^H NMR shows nearly complete conversion (of over 95%). The reaction was washed with saturated Rochelle salt solution (2 × 0.2 L) and brine (0.2 L) while being extracted with 0.2 L of CH_2_Cl_2_. Dried with Na_2_SO_4_, filtered, and then concentrated in vacuo. The crude material was purified through column chromatography (2% EtOAc in hexanes), affording **15** (0.137 kg, 0.47 mol, 93% yield) as a pale-yellow oil. ^1^H NMR (600 MHz, CDCl_3_) δ 3.92–3.83 (m, 1H), 3.58 (ddd, *J* = 7.1, 6.1, 1.5 Hz, 2H), 2.93–2.85 (m, 1H), 2.73 (dd, *J* = 5.1, 4.0 Hz, 1H), 2.44 (dd, *J* = 5.0, 2.7 Hz, 1H), 1.92–1.80 (m, 2H), 1.64–1.38 (m, 6H), 0.87 (s, 9H), 0.06 (d, *J* = 7.4 Hz, 6H). ^13^C{^1^H} NMR (126 MHz, CDCl_3_) δ: 68.9, 52.1, 46.9, 41.7, 39.5, 36.9, 32.5, 25.8, 21.3, 18.0, −4.4, −4.7.

#### (3*S*,7*R*)-7-((*tert*-Butyldimethylsilyl)oxy)-9-chloronon-1-en-3-ol (**16**)

A mixture of trimethylsulfonium iodide (0.185 kg, 0.91 mol, 2.6 equiv) in 0.720 L of THF and 0.260 L of hexanes was cooled to −30 °C before 10 M *n*-BuLi (87 mL, 0.87 mol, 2.5 equiv) was added dropwise by first cannulating into a marked (flame-dried, inert atmosphere, sealed) Erlenmeyer flask and then cannulating the amount into the reaction. After 0.5 h and observing the formation of precipitant, the reaction was warmed to −10 °C before substrate **15** (0.102 kg, 0.35 mol) in solution in 0.150 L of THF was added dropwise. The conditions were maintained for 1 h before warming to ambient temperature. A complete reaction was observed by TLC after 0.5 h at 21 °C. Then, the reaction was quenched with 0.2 L of saturated NH_4_Cl_(aq)_ solution, noticing the color change from colorless to a strong yellow hue, and then washed with 0.2 L of H_2_O and 0.2 L of brine while extracting with 0.2 L of EtOAc. Two back extractions were performed on the combined aqueous layers (2 × 0.2 L) with EtOAc. The organic layers were dried with Na_2_SO_4_, filtered, and then concentrated in vacuo. The crude product was purified by column chromatography (4% EtOAc in hexanes) to give **16** (0.103 kg, 0.34 mol, 96% yield) as a pale-yellow oil. ^1^H and ^13^C NMR spectral data matched those reported in the literature.^5^

#### (3*S*,7*R*)-7-((*tert*-Butyldimethylsilyl)oxy)-9-chloronon-1-en-3-yl Benzoate

A solution of **16** (53 mg, 0.17 mmol) in 1.7 mL of CH_2_Cl_2_ was cooled to 0 °C before pyridine (56 μL, 0.69 mmol, 4.0 equiv) and then benzoyl chloride (61 μL, 0.48 mmol, 2.8 equiv) were added dropwise to the reaction in this order. The solution was warmed to ambient temperature and stirred for 1 h when the reaction was complete by TLC. Then, the solution was quenched with 0.5 mL of H_2_O before washing with H_2_O and extracting with CH_2_Cl_2_; dried with Na_2_SO_4_; filtered; and then concentrated in vacuo. The product was submitted in its crude state to the following reaction.

#### (3*S*,7*R*)-9-Chloro-7-hydroxynon-1-en-3-yl Benzoate

To a solution of (3*S*,7*R*)-7-((tert-butyldimethylsilyl)oxy)-9-chloronon-1-en-3-yl benzoate (74 mg, ∼0.17 mmol) in 9 mL of THF, 4.5 mL of 1 N HCl_(aq)_ was added. The reaction mixture was stirred at an ambient temperature for 16 h and reached completion at this time (confirmed by TLC); washed with saturated NaHCO_3(aq)_ and extracted with EtOAc; dried with Na_2_SO_4_; filtered; and then concentrated in vacuo. The crude material was purified by column chromatography (10% EtOAc in hexanes to 20% EtOAc in hexanes) to give (3*S*,7*R*)-9-chloro-7-hydroxynon-1-en-3-yl benzoate (49 mg, 0.16 mmol, 97% yield over two steps) as a pale-yellow oil. Dr 3:92:5:0 (Chiralcel AD-H analytic; 5% i-PrOH in hexanes; flow rate = 1.0 mL/min; detection at 254 nm; t1 = 15.6 min (minor); t2 = 20.8 min (major); t3 = 22.3 min (minor); t4 = 29.8 min (minor). ^1^H NMR (500 MHz, CDCl_3_) δ 8.06 (dd, 2H), 7.56 (td, *J* = 6.9, 1.4 Hz, 1H), 7.45 (td, 2H), 5.90 (ddd, *J* = 17.0, 10.5, 6.2 Hz, 1H), 5.52 (qt, 1H), 5.34 (dt, *J* = 17.2, 1.3 Hz, 1H), 5.22 (dt, *J* = 10.6, 1.3 Hz, 1H), 3.85 (o, *J* = 8.4, 4.3 Hz, 1H), 3.77–3.61 (m, 2H), 1.95–1.70 (m, 4H), 1.67–1.40 (m, 6H).

#### (3*S*,7*R*)-9-Azido-7-((*tert*-butyldimethylsilyl)oxy)non-1-en-3-ol (**7**)

To a solution of **20** (20.3 g, 66 mmol) in 41 mL of DMF, sodium azide (6.50 g, 100 mmol, 1.5 equiv) and tetrabutylammonium iodide (2.49 g, 6.7 mmol, 0.1 equiv) were added at 21 °C. The mixture was heated to 90 °C. Monitoring by ^1^H NMR showed complete conversion after 7 h. The reaction mixture was cooled to ambient temperature and washed with H_2_O (5 × 20 mL) while extracting 20 mL of a mixture of 20% EtOAc in hexanes. Then, the mixture was dried with MgSO_4_, filtered, and concentrated in vacuo. **7** (20.5 g, 65 mmol, 99% yield) was obtained as a yellow oil. The product was submitted in its crude state to the esterification reactions described below without purification.

#### 2-((4-Methoxybenzyl)oxy)acetic Acid

To a solution of 2-bromoacetic acid (0.505 g, 3.6 mmol) and *para*-methoxybenzyl alcohol (0.45 mL, 3.6 mmol, 1.0 equiv) in 6.4 mL of THF, 60% sodium hydride in mineral oil (0.354 g, 8.9 mmol, 2.4 equiv) was added in three portions at 0 °C. It is observed that the mixture became turbid with the addition of sodium hydride. Then, the mixture was warmed to 21 °C for 20 min before attaching a reflux condenser to the flask (with a drying tube) and heating the reaction to 65 °C, observing the formation of precipitant. Reflux was maintained for 20 h before TLC monitoring indicated a complete reaction. The reaction was quenched with 5 mL of methanol at ambient temperature before concentrating in vacuo; diluted in 5 mL of DI H_2_O; and washed with 5 mL of EtOAc, extracting twice from the organic layer with aqueous extractions. 1 M HCl was added to the aqueous layer until pH ∼ 4 and extracted with 5 mL of CH_2_Cl_2_ five times from the acidic aqueous layer. Organic layers were combined, dried over Na_2_SO_4_, filtered, and concentrated in vacuo to give a yellow oil. The product 2-((4-methoxybenzyl)oxy)acetic acid was submitted in its crude state to the next reaction.

#### 5-Chloro-2-((4-methoxybenzyl)oxy)pentanoic Acid (**20**)

To a solution of diisopropylamine (20.0 mL, 0.14 mol, 2.6 equiv) in 0.180 L of THF, 2.5 M *n*-BuLi (54 mL, 0.14 mmol, 2.5 equiv) was added at −78 °C. After 30 min, a solution of 2-((4-methoxybenzyl)oxy)acetic acid (10.69 g, 54 mmol) in 70 mL of THF was cannulated dropwise into the LDA solution (rinsing the original vessel with THF with 2 × 10 mL). After 1 h at −78 °C, 1-chloro-3-iodopentane (17.5 mL, 0.16 mol, 3.0 equiv) was added (neat) to the reaction. Temperature conditions were maintained for 0.5 h before increasing the bath temperature to −40 °C. TLC monitoring showed complete consumption of starting material after 2 h. The reaction was quenched with 50 mL of 1 M HCl before warming to ambient temperature. The product was extracted with 0.1 L EtOAc and washed with 0.1 L of 1 M HCl; dried over Na_2_SO_4_; filtered; and then concentrated in vacuo. The crude residue was purified by column chromatography (30% EtOAc in hexanes to 50% EtOAc 1% AcOH in hexanes. **20** (7.66 g, 28 mmol, 52% yield over two steps) was obtained as a yellow oil. ^1^H and ^13^C NMR spectral data matched those reported in the literature.^5^

#### (3*S*,7*R*)-9-Azido-7-((*tert*-butyldimethylsilyl)oxy)non-1-en-3-yl 5-Chloro-2-((4-methoxybenzyl)oxy)pentanoate (**21**)

To a solution of **7** (9.12 g, 29 mmol) and **20** (11.9 g, 44 mmol, 1.5 equiv) in 200 mL of CH_2_Cl_2_, 4-dimethylaminopyridine (0.714 g, 5.8 mmol, 0.2 equiv) was added before cooling the reaction to 0 °C. 1-Ethyl-3-(3-dimethylaminopropyl)carbodiimide HCl (11.2 g, 58 mmol, 2.0 equiv) was added in two portions, and then the reaction was warmed to 21 °C after 20 min. TLC monitoring showed a complete reaction after 1 h at ambient temperature. The product was extracted with 50 mL of CH_2_Cl_2_ and washed with 50 mL of saturated NaHCO_3_ aqueous solution. Then, the solution was extracted once more from the aqueous layer with 50 mL of CH_2_Cl_2_. The crude product was purified by column chromatography (2.5% EtOAc in hexanes to 5% EtOAc in hexanes) to give **21** (14.7 g, 26 mmol, 89% yield) as a pale-yellow oil. ^1^H and ^13^C NMR spectral data matched those reported in the literature.^5^

#### (2*S*,9*R*,*E*)-11-Azido-9-((*tert*-butyldimethylsilyl)oxy)-2-(3-chloropropyl)-2-((4-methoxybenzyl)oxy)undec-4-enoic Acid (**22**)

A solution of KN(SiMe_3_)_2_ (9.92 g, 50 mmol, 2.2 equiv) in 80 mL of distilled toluene was cooled to −78 °C for 0.5 h before a solution of **21** (12.8 g, 23 mmol) in 80 mL of distilled toluene was cannulated dropwise into the reaction. After 0.5 h, freshly distilled trimethylsilyl chloride (5.7 mL, 45 mmol, 2.0 equiv) was added dropwise into the reaction mixture. After 1 h, the reaction was warmed to −40 °C; TLC monitoring confirmed a complete reaction after 1 h at this temperature. The reaction was quenched with 0.1 mL of 1 M HCl, and the product was extracted with 0.1 L of EtOAc three times. Organic layers were combined, dried over Na_2_SO_4_, filtered, and concentrated in vacuo. The product was isolated by column chromatography (15% EtOAc in hexanes to 25% EtOAc and 1% AcOH in hexanes) to give **22** (10.5 g, 18 mmol, 82% yield) as a yellow oil. ^1^H and ^13^C NMR spectral data matched those reported in the literature.^5^

#### (2*S*,9*R*,*E*)-11-Azido-9-((*tert*-butyldimethylsilyl)oxy)-2-(3-chloropropyl)-2-((4-methoxybenzyl)oxy)-*N*-((*R*)-1-phenylethyl)undec-4-enamide

1-Ethyl-3-(3-dimethylaminopropyl)carbodiimide HCl (12.7 mg, 0.066 mmol, 3 equiv), (*R*)-(+)-alpha-methylbenzylamine (9 μL, 0.069 mmol, 3 equiv), and HOBt (8.9 mg, 0.053 mmol, 3 equiv) were added sequentially to a solution of acid **22** (12.5 mg, 0.022 mmol) in 0.13 mL of CH_2_Cl_2_. The solution was stirred at room temperature for 1 h and then diluted with 2 mL of CH_2_Cl_2_ and washed with 2 mL of saturated aqueous sodium bicarbonate and then 2 mL of brine. Then, the solution was dried over Na_2_SO_4_, filtered, and concentrated in vacuo. The residue was purified by column chromatography (15% EtOAc in hexanes) to give (2*S*,9*R*,*E*)-11-azido-9-((*tert*-butyldimethylsilyl)oxy)-2-(3-chloropropyl)-2-((4-methoxybenzyl)oxy)-*N*-((*R*)-1-phenylethyl)undec-4-enamide (13.6 mg, 0.20 mmol, 92% yield, dr 14:1) as a clear oil. ^1^H and ^13^C NMR spectral data matched those reported in the literature.^5^

#### 5-Chloropentanoyl Chloride (**18**)

To a solution of 5-chloropentanoic acid (5.90 g, 43 mmol) in 29 mL of CH_2_Cl_2_, oxalyl chloride (4.4 mL, 51 mmol, 1.2 equiv) was added dropwise. The argon line was removed and replaced with a drying tube before a catalytic amount (three drops) of dimethylformamide was added to the reaction. Vigorous bubbling was observed. The reaction was left stirring at an ambient temperature for 2 h while monitoring by ^1^H NMR; complete conversion to 5-chloropentanoyl chloride was observed after 2 h. The reaction was concentrated in vacuo, and the product was distilled under vacuum at 0.2 mmHg (vapor temperature 52–54 °C). **22** (5.87 g, 37 mmol, 88% yield) was obtained as a yellow liquid and submitted to esterification without further purification.

#### (3*S*,7*R*)-9-Azido-7-((*tert*-butyldimethylsilyl)oxy)non-1-en-3-yl 5-Chloropentanoate (**5**)

A solution of **7** (10.0 g, 32 mmol) in 40 mL of CH_2_Cl_2_ was cooled to 0 °C before freshly distilled pyridine (5.2 mL, 64 mmol, 2.0 equiv) was added. A solution of 5-chloropentanoyl chloride (5.87 g, 37 mmol, 1.2 equiv) in 14 mL of CH_2_Cl_2_ (additional 2× 5 mL of CH_2_Cl_2_ to rinse original vessel) was added dropwise to the reaction. A color change from a yellow reaction solution to pink was observed. TLC monitoring indicated a complete reaction after 20 min. The reaction was quenched with 30 mL of 1 N HCl, and the product was extracted with 30 mL of CH_2_Cl_2_ twice. The organic layers were combined, dried with Na_2_SO_4_, filtered, and concentrated under a vacuum. The product was purified by column chromatography (hexanes to 2% EtOAc in hexanes) to provide **5** (13.1 g, 30 mmol, 95% yield) as a pale-yellow oil. ^1^H and ^13^C NMR spectral data matched those reported in the literature.^5^

#### (2*R*,9*R*,*E*)-11-Azido-9-((*tert*-butyldimethylsilyl)oxy)-2-(3-chloropropyl)undec-4-enoic Acid (**23**)

A solution of diisopropylamine (7.2 mL, 51 mmol, 2.17 equiv) in 0.140 L of THF was cooled to −78 °C for 0.5 h before 2.3 M *n*-BuLi (20.5 mL, 47 mmol, 2.0 equiv) was added dropwise. After 0.5 h, a solution of **5** (10.1 g, 23 mmol) in 95 mL of THF was added dropwise to the LDA solution at −78 °C. After another 0.5 h, *tert*-butyldimethylsilyl chloride (8.24 g, 55 mmol, 2.33 equiv) was added, followed by 42 mL of freshly distilled HMPA. The reaction was warmed to −40 °C after 0.5 h and then warmed again to −15 °C after another 0.5 h. The reaction mixture was poured over 15 g of ice and 50 mL of hexanes; the intermediate was extracted with 50 mL of hexanes twice from the aqueous layer. The organic layers were combined, dried over Na_2_SO_4_, filtered, and concentrated in vacuo. The crude intermediate was then diluted in 94 mL of THF and cooled to 0 °C. A 33 mL portion of a prepared 1.8 M aqueous solution of LiOH (cooled to 0 °C for 20 min) was added dropwise to the crude intermediate solution at 0 °C. Monitoring by TLC shows conversion of the intermediate to product after 5 min. The reaction was quenched with 0.15 L of 1 N HCl and extracted with EtOAc. The organic layers were combined, dried over Na_2_SO_4_, filtered, and concentrated in vacuo. The product was purified by column chromatography (7% EtOAc in hexanes to 20% EtOAc and 1% AcOH in hexanes) to provide **23** (8.75 g, 20 mmol, 86% yield) as a yellow oil. ^1^H and ^13^C NMR spectral data matched those reported in the literature.^5^

#### (2*R*,9*R*,*E*)-11-Azido-9-((*tert*-butyldimethylsilyl)oxy)-2-(3-chloropropyl)-*N*-((*R*)-1-phenylethyl)undec-4-enamide

1-Ethyl-3-(3-dimethylaminopropyl)carbodiimide HCl (9.8 mg, 0.051 mmol, 3 equiv), (*R*)-(+)-alpha-methylbenzylamine (7 μL, 0.054 mmol, 3 equiv), and HOBt (6.9 mg, 0.051 mmol, 3 equiv) were added sequentially to a solution of acid **23** (7.4 mg, 0.017 mmol) in 0.10 mL of CH_2_Cl_2_. The solution was stirred at room temperature for 1 h, diluted with 2 mL of CH_2_Cl_2_, and washed with 2 mL of saturated aqueous sodium bicarbonate and then 2 mL of brine. The organic layers were dried over Na_2_SO_4_, filtered, and concentrated in vacuo. The residue was purified by column chromatography (15% EtOAc in hexanes) to give the chiral amide derivative (8.4 mg, 0.16 mmol, 91% yield, dr 7:1) as a clear oil. ^1^H and ^13^C NMR spectral data matched those reported in the literature.^5^

#### Allyl (2*R*,9*R*,*E*)-11-Azido-9-((*tert*-butyldimethylsilyl)oxy)-2-(3-chloropropyl)undec-4-enoate

To a solution of **23** (7.76 g, 18 mmol) in 36 mL of distilled dimethylformamide, K_2_CO_3_ (5.46 g, 39 mmol, 2.2 equiv) was added at 21 °C and then freshly distilled allyl bromide (4.7 mL, 54 mmol, 3.0 equiv) was added dropwise to the reaction. Monitoring by TLC showed a complete reaction after 6 h. The reaction was diluted in 30 mL of hexanes and quenched with slow addition of 10 mL of 1 N HCl. The product was extracted with 20 mL of hexanes and washed with 15 mL of DI H_2_O three times. The organic layer was dried in Na_2_SO_4_, filtered, and concentrated in vacuo. The crude material was purified by column chromatography (4% EtOAc in hexanes) to obtain allyl (2*R*,9*R*,*E*)-11-azido-9-((*tert*-butyldimethylsilyl)oxy)-2-(3-chloropropyl)undec-4-enoate (7.23 g, 15 mmol, 85% yield) as a clear oil. ^1^H NMR (600 MHz, CDCl_3_) δ 5.89 (ddt, *J* = 17.3, 10.4, 5.8 Hz, 1H), 5.43 (dtt, *J* = 14.6, 6.6, 1.2 Hz, 1H), 5.38–5.26 (m, 2H), 5.22 (dq, *J* = 10.4, 1.3 Hz, 1H), 4.56 (dq, *J* = 5.7, 1.4 Hz, 2H), 3.75 (dtd, *J* = 7.2, 5.6, 4.3 Hz, 1H), 3.59–3.43 (m, 2H), 3.41–3.25 (m, 2H), 2.48–2.38 (m, 1H), 2.36–2.24 (m, 1H), 2.24–2.12 (m, 1H), 1.96 (q, *J* = 6.9 Hz, 2H), 1.88–1.59 (m, 6H), 1.49–1.26 (m, 4H), 0.87 (d, *J* = 1.4 Hz, 9H). ^13^C{^1^H} NMR (126 MHz, CDCl_3_) δ: 174.9, 133.0, 132.4, 126.8, 118.3, 118.3, 69.3, 65.0, 48.1, 45.2, 44.7, 36.8, 35.7, 35.4, 32.6, 30.4, 28.9, 26.0, 24.9, 18.1, −4.3, −4.6. HRMS (ESI) *m*/*z*: [M+Na^+^] calcd for (C_23_H_42_ClN_3_O_3_SiNa^+^) 494.2582; found 494.2577.

#### Allyl (2*R*,9*R*,*E*)-11-Amino-9-((*tert*-butyldimethylsilyl)oxy)-2-(3-chloropropyl)undec-4-enoate (**24**)

A 100 mL round-bottom flask was charged with a stirbar, gas inlet adapter, and septum before it was flame-dried under vacuum and backfilled with argon. The flask was charged with SnCl_2_ (4.12 g, 22 mmol, 1.5 equiv) under nitrogen and then reconnected to an argon line. 8.7 mL of distilled acetonitrile was added before the mixture was cooled to 0 °C. Thiophenol (6.6 mL, 65 mmol, 4.5 equiv) was added dropwise to the reaction, followed by dropwise addition of distilled triethylamine (9.1 mL, 65 mmol, 4.5 equiv). The reaction's color change from colorless to bright yellow was observed. The reaction was removed from the cold bath to reach ambient temperature for 15 min, after which the reaction was cooled back down to 0 °C again. A solution of allyl (2*R*,9*R*,*E*)-11-azido-9-((*tert*-butyldimethylsilyl)oxy)-2-(3-chloropropyl)undec-4-enoate (6.82 g, 14 mmol) in 2.0 mL of distilled acetonitrile (and 2× 1.9 mL rinses of the original vessel) was added dropwise to the reaction at 0 °C. Once the substrate solution had been completely included in the reaction, the reaction mixture was removed from the cold bath and allowed to reach ambient temperature. The reaction's color change from yellow to orange as well as gas evolution was observed. The bubbling stopped after 15 min, and the reaction was then diluted in 0.1 L of CH_2_Cl_2_ and washed with 50 mL of 3 M NaOH twice. The organic layer was dried over Na_2_SO_4_, filtered, and concentrated in vacuo. Crude ^1^H NMR shows a complete conversion of the azide substrate to the amine product. The crude material was purified by column chromatography to remove thiophenol (20% EtOAc in hexanes to 20% MeOH and 1% NH_4_OH in CH_2_Cl_2_) to afford **24** (6.45 g, 14 mmol, 100% yield) as a light-brown oil. ^1^H NMR (600 MHz, CDCl_3_) δ 5.93–5.82 (m, 1H), 5.48–5.37 (m, 1H), 5.36–5.24 (m, 2H), 5.21 (dt, *J* = 10.3, 1.2 Hz, 1H), 4.55 (dt, *J* = 5.9, 1.4 Hz, 2H), 3.71 (p, *J* = 5.7 Hz, 1H), 3.49 (td, *J* = 6.3, 2.7 Hz, 2H), 2.73 (hept, *J* = 6.3 Hz, 2H), 2.45–2.35 (m, 1H), 2.30 (dt, *J* = 14.4, 7.5 Hz, 1H), 2.17 (dt, *J* = 13.8, 6.7 Hz, 1H), 1.94 (t, *J* = 7.0 Hz, 4H), 1.80–1.60 (m, 4H), 1.57 (h, *J* = 6.4 Hz, 2H), 1.44–1.36 (m, 2H), 1.36–1.27 (m, 2H), 0.89–0.83 (m, 9H), 0.02 (d, *J* = 4.0 Hz, 6H). ^13^C{^1^H} NMR (126 MHz, CDCl_3_) δ: 174.9, 133.2, 132.3, 126.6, 118.3, 70.5, 65.0, 45.2, 44.7, 40.4, 38.7, 36.9, 35.4, 32.7, 30.4, 28.9, 26.0, 25.0, 18.2, −4.3, −4.4. HRMS (ESI) *m*/*z*: [M+H^+^] calcd for (C_23_H_45_ClNO_3_Si) 446.2587; found 446.2584.

#### Allyl (2*R*,9*R*,*E*)-11-((2*S*,9*R*,*E*)-11-Azido-9-((*tert*-butyldimethylsilyl)oxy)-2-(3-chloropropyl)-2-((4-methoxybenzyl)oxy)undec-4-enamido)-9-((*tert*-butyldimethylsilyl)oxy)-2-(3-chloropropyl)undec-4-enoate (**25**)

To a mixture of **22** (9.04 g, 16 mmol, 1.1 equiv) and **24** (6.45 g, 14 mmol) in 15.2 mL of CH_2_Cl_2_ at 21 °C, HOBt (2.93 g, 22 mmol, 1.5 equiv) was added, followed by 1-ethyl-3-(3-dimethylaminopropyl)carbodiimide HCl (4.16 g, 22 mmol, 1.5 equiv). Monitoring by TLC showed consumption of **24** after 0.5 h. The reaction was diluted in 40 mL of 50% EtOAc in hexanes and quenched with 30 mL of saturated NaHCO_3(aq)_ and was washed with 30 mL of brine. Back-extraction was performed using 30 mL of 50% EtOAc in hexanes to extract from the combined aqueous layers. The organic layers were combined, dried over Na_2_SO_4_, filtered, and concentrated in vacuo. The crude product was purified by column chromatography (7.5% EtOAc in hexanes to 20% EtOAc in hexanes) to give **25** (11.2 g, 11 mmol, 78% yield) as a pale-yellow oil. ^1^H NMR (600 MHz, CDCl_3_) δ 7.25 (s, 3H), 6.92–6.87 (m, 2H), 6.83 (t, *J* = 6.0 Hz, 1H), 5.90 (ddt, *J* = 17.2, 10.4, 5.7 Hz, 1H), 5.56–5.48 (m, 1H), 5.47–5.39 (m, 1H), 5.35–5.27 (m, 3H), 5.23 (dq, *J* = 10.4, 1.3 Hz, 1H), 4.57 (dt, *J* = 5.8, 1.4 Hz, 2H), 4.42 (d, *J* = 9.9 Hz, 1H), 4.34 (d, *J* = 9.9 Hz, 1H), 3.82 (s, 3H), 3.79–3.72 (m, 1H), 3.68–3.55 (m, 2H), 3.51 (td, *J* = 6.3, 2.4 Hz, 2H), 3.49–3.42 (m, 1H), 3.33 (qt, *J* = 10.4, 3.8 Hz, 3H), 3.23–3.13 (m, 1H), 2.63 (dd, *J* = 15.0, 6.5 Hz, 1H), 2.54 (dd, *J* = 15.0, 7.4 Hz, 1H), 2.43 (ddd, *J* = 11.5, 7.9, 5.7 Hz, 1H), 2.32 (dt, *J* = 14.3, 7.3 Hz, 1H), 2.18 (dt, *J* = 13.7, 6.7 Hz, 1H), 2.06–1.97 (m, 3H), 1.97–1.90 (m, 3H), 1.83–1.47 (m, 13H), 1.46–1.23 (m, 11H), 0.91–0.82 (m, 18H), 0.05 (d, *J* = 5.6 Hz, 6H), −0.02 (d, *J* = 15.3 Hz, 6H). ^13^C{^1^H} NMR (126 MHz, CDCl_3_) δ: 175.0, 172.8, 159.5, 134.0, 133.2, 132.4, 130.0, 129.6, 126.7, 124.1, 118.4, 114.1, 83.0, 70.1, 69.3, 65.1, 63.9, 55.4, 48.1, 45.2, 45.2, 44.8, 38.3, 36.9, 36.9, 36.8, 35.9, 35.8, 35.4, 32.9, 32.7, 31.8, 30.4, 28.9, 26.9, 26.0, 25.0, 24.9, 18.2, −4.2, −4.2, −4.5, −4.5. HRMS (ESI) *m*/*z*: [M+Na^+^] calcd for (C_51_H_88_Cl_2_N_4_O_7_Si_2_Na^+^) 1017.5466; found 1017.5464.

#### Allyl (2*R*,9*R*,*E*)-11-((2*S*,9*R*,*E*)-11-Amino-9-((*tert*-butyldimethylsilyl)oxy)-2-(3-chloropropyl)-2-((4-methoxybenzyl)oxy)undec-4-enamido)-9-((*tert*-butyldimethylsilyl)oxy)-2-(3-chloropropyl)undec-4-enoate (**26**)

A 250 mL round-bottom flask was charged with a stirbar, gas inlet adapter, and septum before it was flame-dried under vacuum and backfilled with argon. The flask was charged with SnCl_2_ (2.53 g, 13 mmol, 1.19 equiv) under nitrogen and then reconnected to an argon line. 6.7 mL of distilled acetonitrile was added before the mixture was cooled to 0 °C. Thiophenol (4.1 mL, 40 mmol, 3.57 equiv) was added dropwise to the reaction, followed by dropwise addition of distilled triethylamine (5.6 mL, 40 mmol, 3.57 equiv). The reaction's color change from colorless to bright yellow was observed. The reaction was removed from the cold bath to reach ambient temperature for 15 min, after which the reaction was cooled back down to 0 °C again. A solution of **25** (11.2 g, 11 mmol) in 4.5 mL of distilled acetonitrile was added dropwise to the reaction mixture at 0 °C. Once the substrate solution had been completely included in the reaction, the reaction mixture was removed from the cold bath and allowed to reach ambient temperature. The reaction's color change from yellow to orange, as well as gas evolution, was observed. The bubbling stopped after 0.5 h, and the reaction was then diluted in 100 mL of CH_2_Cl_2_ and washed with 50 mL of NaOH twice. The organic layer was dried over Na_2_SO_4_, filtered, and concentrated in vacuo. Crude ^1^H NMR shows a complete conversion of the azide substrate to the amine product. The crude material was purified by column chromatography to remove thiophenol (20% EtOAc in hexanes to 25% MeOH and 2% NH_4_OH in CH_2_Cl_2_), affording **26** (10.9 g, 11 mmol, 100% yield) as a light-brown oil. ^1^H NMR (600 MHz, CDCl_3_) δ 7.25 (d, *J* = 6.9 Hz, 2H), 6.92–6.87 (m, 2H), 6.84 (t, *J* = 6.0 Hz, 1H), 5.95–5.84 (m, 1H), 5.50 (dt, *J* = 14.4, 6.8 Hz, 1H), 5.43 (dt, *J* = 14.0, 6.7 Hz, 1H), 5.37–5.26 (m, 3H), 5.23 (dq, *J* = 10.4, 1.3 Hz, 1H), 4.57 (dt, *J* = 5.8, 1.4 Hz, 2H), 4.42 (d, *J* = 10.0 Hz, 1H), 4.34 (d, *J* = 9.9 Hz, 1H), 3.82 (s, 4H), 3.78–3.71 (m, 2H), 3.68–3.60 (m, 2H), 3.60–3.53 (m, 1H), 3.51 (dt, *J* = 6.4, 3.1 Hz, 2H), 3.45 (ddd, *J* = 10.5, 8.5, 5.6 Hz, 1H), 3.34 (ddd, *J* = 13.8, 8.2, 4.2 Hz, 1H), 3.21–3.11 (m, 1H), 2.84 (t, *J* = 7.2 Hz, 2H), 2.62 (dd, *J* = 15.0, 6.6 Hz, 1H), 2.54 (dd, *J* = 15.0, 7.4 Hz, 1H), 2.43 (tt, *J* = 7.7, 5.5 Hz, 1H), 2.32 (td, *J* = 14.1, 7.0 Hz, 1H), 2.18 (dt, *J* = 13.7, 6.8 Hz, 1H), 2.03–1.91 (m, 8H), 1.82–1.58 (m, 11H), 1.51 (dtd, *J* = 13.2, 7.7, 5.4 Hz, 2H), 1.41 (ddt, *J* = 16.0, 10.8, 5.6 Hz, 5H), 1.37–1.21 (m, 9H), 0.91–0.86 (m, 13H), 0.84 (d, *J* = 2.8 Hz, 11H), 0.05 (s, 7H), −0.02 (d, *J* = 16.1 Hz, 6H). ^13^C{^1^H} NMR (126 MHz, CDCl_3_) δ: 174.9, 172.7, 159.4, 134.0, 133.1, 133.0, 132.3, 129.9, 129.5, 126.6, 123.9, 118.3, 118.2, 113.9, 82.9, 77.4, 77.4, 77.2, 77.1, 76.9, 70.4, 70.0, 65.0, 65.0, 63.8, 55.3, 55.3, 45.1, 45.1, 45.0, 44.7, 44.6, 39.5, 38.4, 38.2, 36.8, 36.8, 36.6, 35.8, 35.3, 32.9, 32.6, 31.7, 30.3, 28.8, 26.8, 25.9, 25.9, 25.0, 24.9, 18.1, 18.1, 18.1, −4.3, −4.3, −4.5, −4.5. HRMS (ESI) *m*/*z*: [M + H] calcd for (C_51_H_91_Cl_2_N_2_O_7_Si_2_^+^) 969.5742; found 969.5719.

#### (3*S*,5*E*,10*R*,15*R*,17*E*,22*R*)-10,22-Bis((*tert*-butyldimethylsilyl)oxy)-3,15-bis(3-chloropropyl)-3-((4-methoxybenzyl)oxy)-1,13-diazacyclotetracosa-5,17-diene-2,14-dione (**27**)

To a solution of **26** (2.08 g, 2.1 mmol) in 2.7 mL of CH_2_Cl_2_, PhSiH_3_ (0.79 mL, 6.4 mmol, 3.0 equiv) was added dropwise. Pd(PPh_3_)_4_ (74.4 mg, 0.064 mmol, 0.03 equiv) was added (charged flame-dried dram vial under a N_2_ environment). The reaction mixture rapidly changed color from light brown to black. After 0.5 h, TLC monitoring indicated consumption of starting material and the reaction mixture was concentrated in vacuo, and then column chromatography was rapidly performed to purify it (25% EtOAc in hexanes to 25% MeOH 1% NH_4_OH in CH_2_Cl_2_) to provide a brown foam. The product was submitted rapidly to the next reaction in the synthesis.

To a solution of (2*R*,9*R*,*E*)-11-((2*S*,9*R*,*E*)-11-amino-9-((*tert*-butyldimethylsilyl)oxy)-2-(3-chloropropyl)-2-((4-methoxybenzyl)oxy)undec-4-enamido)-9-((*tert*-butyldimethylsilyl)oxy)-2-(3-chloropropyl)undec-4-enoic acid (1.97 g, ∼2.1 mmol) in 0.7 L of DMF, ethyl diisopropylamine (1.5 mL, 8.6 mmol, 4.0 equiv) was added, followed by HATU (1.21 g, 3.2 mmol, 1.5 equiv). The reaction was stirred at an ambient temperature for 3 days with monitoring by ^1^H NMR. When the reaction had reached its extent, the reaction was quenched with 5 mL of DI H_2_O and the DMF was removed by distillation in vacuo (0.2 mmHg, rotovap bath set to 50 °C). The crude reaction mixture was washed with 0.1 L of water five times and the product was extracted by 0.1 L of EtOAc. The organic layer was dried over Na_2_SO_4_, filtered, and concentrated in vacuo. The product was isolated by column chromatography (25% EtOAc in hexanes to 40% EtOAc in hexanes) to afford **27** (0.602 g, 0.66 mmol, 31% yield over two steps) as a white solid. ^1^H NMR (600 MHz, CDCl_3_) δ 7.26 (d, *J* = 8.8 Hz, 4H), 6.93 (dd, *J* = 7.8, 4.4 Hz, 1H), 6.90 (d, 2H), 6.27 (t, *J* = 4.9 Hz, 1H), 5.52 (dt, *J* = 15.3, 6.5 Hz, 1H), 5.44 (dt, *J* = 15.1, 6.4 Hz, 1H), 5.35–5.24 (m, 2H), 4.43 (d, *J* = 10.2 Hz, 1H), 4.36 (d, *J* = 10.1 Hz, 1H), 3.82 (s, 3H), 3.81 (s, 1H), 3.73 (dq, *J* = 10.2, 5.5 Hz, 1H), 3.59 (dt, *J* = 11.1, 5.7 Hz, 1H), 3.52 (dt, *J* = 8.1, 6.0 Hz, 2H), 3.50–3.47 (m, 1H), 3.47–3.35 (m, 2H), 3.21 (dq, *J* = 11.5, 5.3 Hz, 1H), 3.06–2.98 (m, 1H), 2.59 (dd, *J* = 14.9, 7.2 Hz, 1H), 2.53 (dd, *J* = 14.8, 6.9 Hz, 1H), 2.27 (ddd, *J* = 13.7, 10.8, 7.5 Hz, 1H), 2.10 (dt, *J* = 13.0, 5.1 Hz, 1H), 2.05–1.93 (m, 5H), 1.77 (tq, *J* = 9.2, 4.6 Hz, 3H), 1.74–1.64 (m, 4H), 1.58–1.51 (m, 2H), 1.51–1.35 (m, 6H), 1.32 (p, *J* = 7.8 Hz, 4H), 1.25 (d, *J* = 2.3 Hz, 1H), 0.88 (s, 9H), 0.84 (s, 9H), 0.10 – −0.05 (m, 12H). ^13^C{^1^H} NMR (126 MHz, CDCl_3_) δ: 174.4, 172.6, 159.3, 133.8, 132.6, 130.2, 129.4, 127.0, 123.7, 114.1, 83.3, 71.5, 70.7, 63.7, 55.5, 48.1, 45.2, 45.0, 38.3, 37.7, 36.8, 36.6, 36.5, 36.4, 36.2, 35.0, 33.1, 33.0, 31.6, 30.9, 30.3, 26.9, 26.1, 26.0, 26.0, 25.0, 24.8, 18.2, 18.1, −4.2, −4.4, −4.4. HRMS(ESI) *m*/*z*: [M + H] calcd for (C_48_H_85_Cl_2_N_2_O_6_Si_2_^+^) 911.5324; found 911.5339.

#### (4*R*,8*E*,11*S*,18*R*,22*E*,25*R*)-4,18-Bis((*tert*-butyldimethylsilyl)oxy)-11-((4-methoxybenzyl)oxy)-1,15-diazatricyclo[23.3.1.1^11,15^]triaconta-8,22-diene-29,30-dione (**28**)

A solution of LiN(SiMe_3_)_2_ was prepared by adding a 2.35 M solution of *n*-BuLi (5.3 mL, 12 mmol) to a solution of freshly distilled bis(trimethylsilyl)amine (2.8 mL, 13 mmol) in 25 mL of distilled THF at 0 °C. After 15 min, the reaction was removed from the cold bath and allowed to reach ambient temperature.

To a solution of **27** (2.81 g, 3.1 mmol) in 15.5 mL of THF, 0.1 equiv of LiN(SiMe_3_)_2_ was added at a time until 2.3 equiv (14.2 mL) of 0.5 M LiN(SiMe_3_)_2_ solution was added to the reaction at 21 °C. TLC analysis showed near-complete conversion of this intermediate at this time. More LiN(SiMe_3_)_2_ solution was not added to prevent epimerization with excess LiN(SiMe_3_)_2_. The reaction was quenched with 10 mL of saturated NH_4_Cl_(aq)_ solution, and the crude product was extracted with 20 mL of EtOAc and washed with 20 mL of DI H_2_O. The crude solution was dried over Na_2_SO_4_, filtered, and concentrated in vacuo. The product was isolated by column chromatography (17% EtOAc in hexanes to 30% EtOAc in hexanes) to give **28** (2.10 g, 2.5 mmol, 81% yield). ^1^H NMR (600 MHz, CDCl_3_) δ 7.24 (d, *J* = 8.1 Hz, 2H), 6.84 (d, *J* = 8.2 Hz, 2H), 5.55–5.36 (m, 3H), 5.27 (dt, *J* = 15.0, 7.3 Hz, 1H), 4.59 (d, *J* = 11.1 Hz, 1H), 3.87–3.80 (m, 1H), 3.78 (s, 4H), 3.69 (hept, *J* = 5.6 Hz, 2H), 3.43 (q, *J* = 6.8 Hz, 1H), 3.36 (dt, *J* = 14.0, 7.1 Hz, 1H), 3.29 (td, *J* = 11.2, 4.9 Hz, 1H), 3.21 (dt, *J* = 17.4, 6.3 Hz, 2H), 3.01 (ddd, *J* = 17.2, 13.2, 7.5 Hz, 2H), 2.42 (dt, *J* = 13.2, 6.5 Hz, 1H), 2.38–2.28 (m, 2H), 2.19 (dd, *J* = 13.1, 8.3 Hz, 1H), 2.15–2.06 (m, 1H), 1.97 (qd, *J* = 14.9, 6.6 Hz, 6H), 1.90–1.82 (m, 4H), 1.67–1.54 (m, 5H), 1.50 (qd, *J* = 8.9, 4.1 Hz, 1H), 1.40 (ttd, *J* = 15.5, 11.3, 4.5 Hz, 7H), 1.34–1.24 (m, 9H), 0.89 (d, *J* = 2.4 Hz, 18H), 0.06 (d, *J* = 2.6 Hz, 12H). ^13^C{^1^H} NMR (126 MHz, CDCl_3_) δ: 171.8, 168.5, 159.0, 134.6, 132.5, 131.7, 129.2, 129.1, 128.1, 125.2, 113.8, 113.8, 77.5, 70.8, 70.4, 65.8, 65.8, 55.4, 48.8, 48.7, 48.3, 44.1, 44.1, 42.0, 39.1, 36.5, 35.8, 35.2, 34.8, 34.7, 34.2, 33.9, 32.7, 32.2, 32.1, 31.7, 29.8, 29.2, 26.6, 26.0, 25.4, 25.3, 25.2, 22.8, 22.8, 22.3, 20.8, 19.0, 18.2, 14.3, 11.6, −4.2, −4.2, −4.2, −4.3, −4.4, −4.4. HRMS (ESI) *m*/*z*: [M+Na^+^] calcd for (C_48_H_82_N_2_O_6_Si_2_Na^+^) 861.5609; found 861.5635.

#### (4*R*,8*E*,11*S*,18*R*,22*E*,25*R*)-4,18-Dihydroxy-11-((4-methoxybenzyl)oxy)-1,15-diazatricyclo[23.3.1.1^11,15^]triaconta-8,22-diene-29,30-dione

A 1 M solution of tetrabutylammonium fluoride trihydrate solution (10.1 mL, 10 mmol, 4.0 equiv) was added to **28** (2.10 g, 2.5 mmol) at 0 °C. The reaction was then warmed to ambient temperature and then heated to 50 °C for 3 h. TLC analysis showed complete consumption of starting material. The reaction was quenched with 4 mL of a saturated solution of NH_4_Cl and then extracted with 10 mL of EtOAc while washing with 10 mL of brine (five times). The organic layer was dried over Na_2_SO_4_, filtered, and concentrated in vacuo. The crude product was purified using a silica plug (70% acetone in hexanes) to provide (4*R*,8*E*,11*S*,18*R*,22*E*,25*R*)-4,18-dihydroxy-11-((4-methoxybenzyl)oxy)-1,15-diazatricyclo[23.3.1.1^11,15^]triaconta-8,22-diene-29,30-dione. The product was submitted to the following reaction as a mixture, with *tert-*butyldimethylsilanol being the significant impurity.

#### (4*R*,8*E*,11*S*,18*R*,22*E*,25*R*)-4,11,18-Trihydroxy-1,15-diazatricyclo[23.3.1.111,15]triaconta-8,22-diene-29,30-dione (**29**)

To a mixture of (4*R*,8*E*,11*S*,18*R*,22*E*,25*R*)-4,18-dihydroxy-11-((4-methoxybenzyl)oxy)-1,15-diazatricyclo[23.3.1.1^11,15^]triaconta-8,22-diene-29,30-dione (1.55 g, 2.5 mmol), 25.4 mL of distilled CH_2_Cl_2_, and 2.5 mL of DI H_2_O, 2,3-dichloro-5,6-dicyano-*p*-benzoquinone (0.724 g, 3.2 mmol, 1.25 equiv) were added in one portion at ambient temperature. The heterogeneous mixture's color change from light yellow to fuchsia was observed. TLC monitoring indicated complete consumption of starting material after 10 min. The reaction was quenched with 20 mL of an aqueous mixture of 50% saturated aqueous NaHCO_3_ and 50% brine; the crude product was extracted with 30 mL of CH_2_Cl_2_. The organic layer was dried with Na_2_SO_4_, filtered, and concentrated in vacuo. The product was isolated by column chromatography (30% hexanes in EtOAc to 10% MeOH in CH_2_Cl_2_) to obtain **29** (1.07 g, 2.2 mmol, 85% over two steps) as an off-white solid. ^1^H NMR (600 MHz, CDCl_3_) δ 5.53 (dt, *J* = 14.1, 6.5 Hz, 1H), 5.45–5.38 (m, 2H), 5.36 (dd, *J* = 15.2, 7.6 Hz, 1H), 3.82 (ddd, *J* = 14.1, 9.5, 4.8 Hz, 1H), 3.63 (s, 1H), 3.58 (ddd, *J* = 14.2, 9.0, 5.6 Hz, 1H), 3.46 (s, 1H), 3.42 (s, 1H), 3.37–3.29 (m, 3H), 3.26 (dd, *J* = 7.9, 4.3 Hz, 3H), 3.08 (dt, *J* = 13.9, 5.2 Hz, 1H), 2.42 (td, *J* = 12.5, 6.6 Hz, 2H), 2.35 (dd, *J* = 13.2, 7.3 Hz, 2H), 2.29 (dt, *J* = 12.4, 6.0 Hz, 1H), 2.13–2.04 (m, 3H), 2.03–1.96 (m, 3H), 1.94 (s, 1H), 1.92–1.83 (m, 4H), 1.80–1.58 (m, 6H), 1.54–1.45 (m, 10H). HRMS (ESI) *m*/*z*: [M+Na^+^] calcd for (C_28_H_46_N_2_O_5_Na^+^) 513.3304; found 513.3298.

#### (4*aS*,6*E*,11*R*,12*aS*,16*aR*,18*E*,23*R*,24*aS*)-3,4,8,9,10,11,12*a*,15,16,16*a*,20,21,22,23-Tetradecahydro-2*H*,5*H*,14*H*,17*H*-1,23:11,13-diethano[1,11]dioxacycloicosino[2,3-*b*:12,13-*b*′]dipyridin-4*a*(24*aH*)-ol

A 100 mL round-bottom flask and a 250 mL round-bottom flask (charged with a glass stirbar) were dried under vacuum and backfilled with argon. Both flasks were cooled to −78 °C. NH_3_ was condensed from a cylinder into the 100 mL round-bottom flask, marked to 50 mL, before it was cannulated into the precooled 250 mL round-bottom flask. Lithium metal was added in six portions amounting to 0.373 g (54 mmol, ∼30 equiv) in total, and the solution was observed to change from colorless to a deep-blue color. The reaction was warmed to −40 °C for 1 h. After this, the solution was again cooled to −78 °C for 20 min, before 2 mL of THF was added. The substrate **33** (0.869 g, 1.8 mmol) was prepared as a solution in 30 mL of THF (and 3 mL to rinse the original container and syringe), which was added dropwise to the reaction at −78 °C. The reaction was kept at −78 °C for 15 min before warming to −40 °C and maintaining the temperature for 30 min. After this time, the reaction was quenched with the addition of 5 g NH_4_Cl_(s)_, followed by 30 mL of THF and warming to 21 °C for 20 min. 5 mL of DI H_2_O was added before 30 mL CH_2_Cl_2_ was included. The reaction was washed with 20 mL of 1 M NaOH_(aq)_ and extracted with 10 mL of CH_2_Cl_2_. The crude product material was submitted to column chromatography (reverse-phase silica gel) (10% DI H_2_O in MeOH to 5% DI H_2_O in MeOH) and isolated as a mixture of three products weighing 0.606 g as a white semisolid. The material was submitted as a mixture to the final reaction in the synthesis.

#### (+)-Desmethylxestospongin B (**1**)

The product mixture from the previous reaction (0.606 g) was dissolved in HPLC-grade EtOAc. 5 wt % rhodium on alumina (0.304 g) was added to the solution, and the reaction vessel was sealed. A balloon filled with H_2_ was fixed to a syringe with needle and was introduced through the septum of the reaction vessel. An outlet needle was introduced, and H_2_ was allowed to bubble through the solution for 5 min, after which the outlet needle was removed and the H_2_ source needle was placed above the reaction solution. Every 30 min, H_2_ was bubbled through the reaction mixture for 5 min and the reaction was monitored by ^1^H NMR; after 2 h, the reaction was deemed complete. The reaction mixture was filtered through Celite (filtering agent was rinsed with HPLC-grade EtOAc) and concentrated in vacuo. The product was purified via column chromatography (reverse-phase silica gel) (10% H_2_O in MeOH to 5% H_2_O in MeOH) to give **1** (0.366 g, 0.79 mmol, 45% yield over two steps) as a white solid. ^1^H and ^13^C NMR spectral data matched those reported in the literature.^5^

## Data Availability

The data underlying this study are available in the published article and its Supporting Information.

## Supporting Information Available

Copies of NMR spectra (PDF)
