# Supplementary material for: Scalable Total Synthesis of (+)-Desmethylxestospongin B
Source: J Org Chem. 2024 May 29;89(11):8120–30. doi: 10.1021/acs.joc.4c00779 (PMC11165571; doi:10.1021/acs.joc.4c00779)
Supplement: Supplementary file 1 — jo4c00779_si_001.pdf [file jo4c00779_si_001.pdf]

Supporting Information

Scalable Total Synthesis of (+)-Desmethylxestospongin B

Alana K. Borum, Karen Y. Chen, Armen Zakarian*

Department of Chemistry and Biochemistry, University of California, Santa Barbara, California 93106, United States.

zakarian@chem.ucsb.edu

Supplementary Information I

^{1}H NMR (600 MHz, $CDCl_3$) and $^{13}C\{^1H\}$ NMR (126 MHz, $CDCl_3$) spectrum of intermediate 13

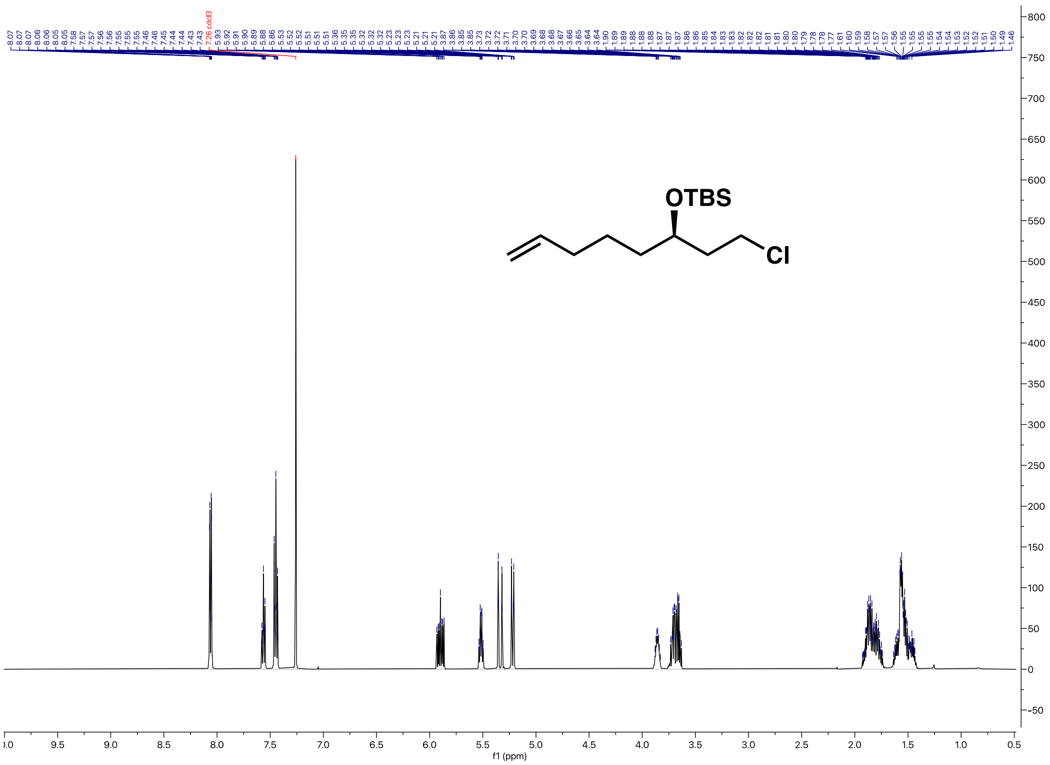

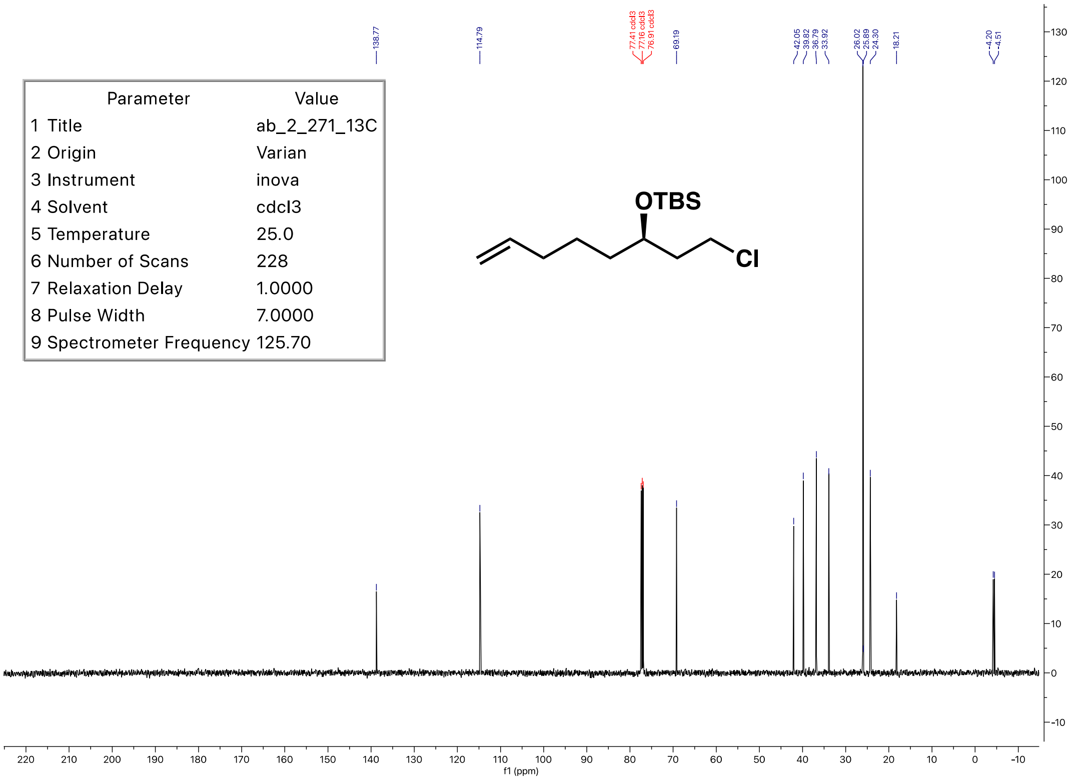
Parameter Value
1 Title ab_2_271_13C
2 Origin Varian
3 Instrument inova
4 Solvent cdcl3
5 Temperature 25.0
6 Number of Scans 228
7 Relaxation Delay 1.0000
8 Pulse Width 7.0000
9 Spectrometer Frequency 125.70
OTBS
Cl
138.77
114.79
77.41 cdcl3
77.16 cdcl3
76.91 cdcl3
69.19
42.05
39.82
36.79
33.92
26.02
25.89
24.30
18.21
-4.20
-4.51
f1 (ppm)

^{1}H NMR (600 MHz, $CDCl_3$) and $^{13}C\{^{1}H\}$ NMR (126 MHz, $CDCl_3$) spectrum of intermediate 15

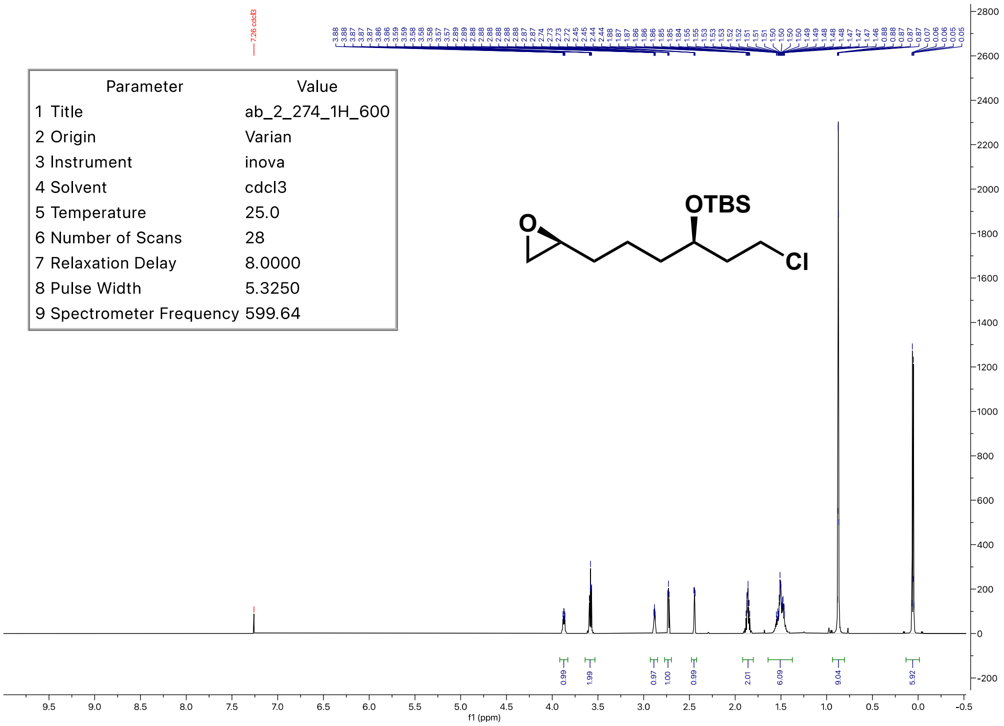

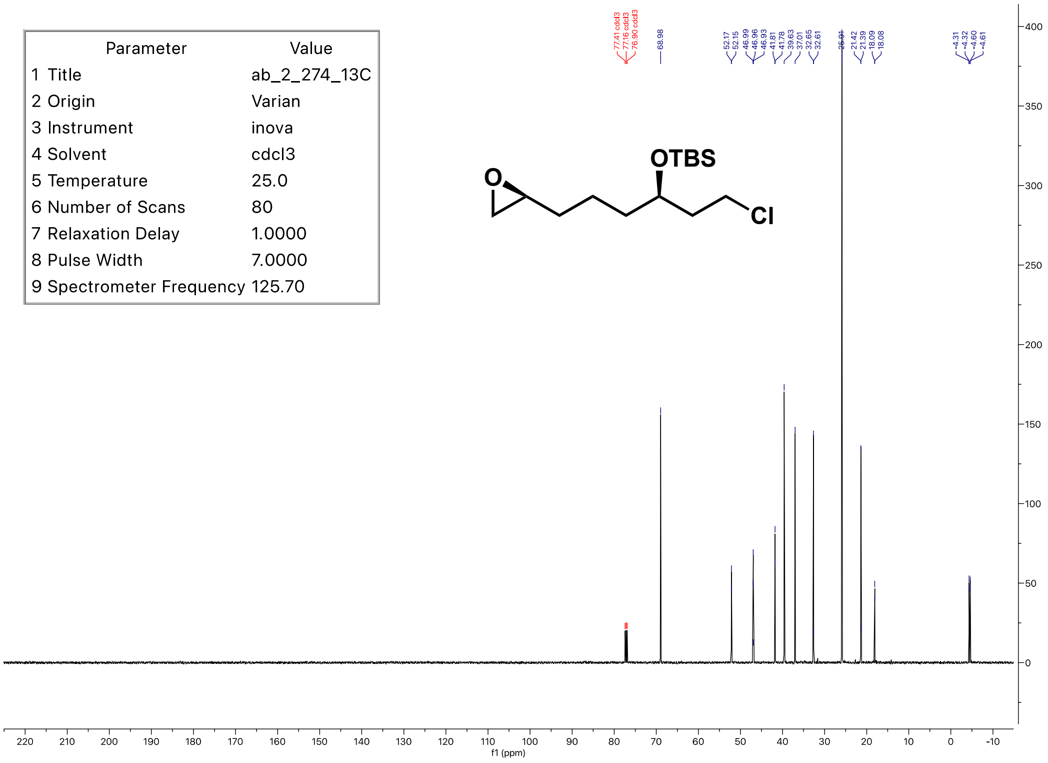

^{1}H NMR (600 MHz, $CDCl_3$) and $^{13}C\{^{1}H\}$ NMR (126 MHz, $CDCl_3$) spectrum of derivative of 15

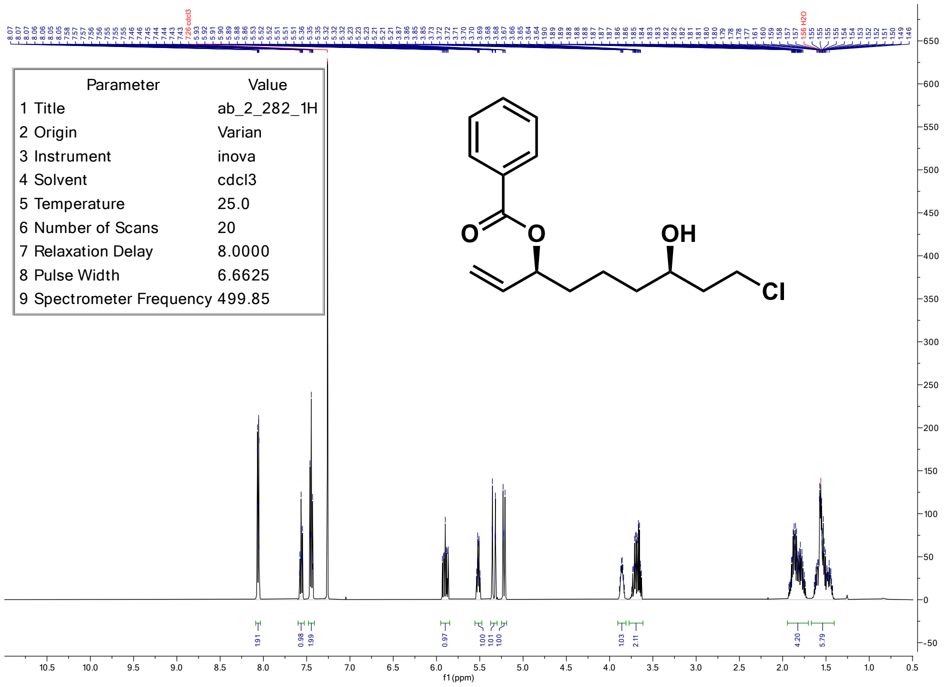

SHIMADZU LabSolutions

Analysis Report

<Sample Information>

Sample Name	: akb_2_282		
Sample ID	: akb_2_282		
Data Filename	: akb_2_282.lcd		
Method Filename	: JakeAnalytical.lcm		
Batch Filename	:		
Vial #	: 1-1	Sample Type	: Unknown
Injection Volume	: 1 uL		
Date Acquired	: 4/7/2023 12:09:49 PM	Acquired by	: Alana Borum
Date Processed	: 4/7/2023 12:44:56 PM	Processed by	: Alana Borum

<Chromatogram>

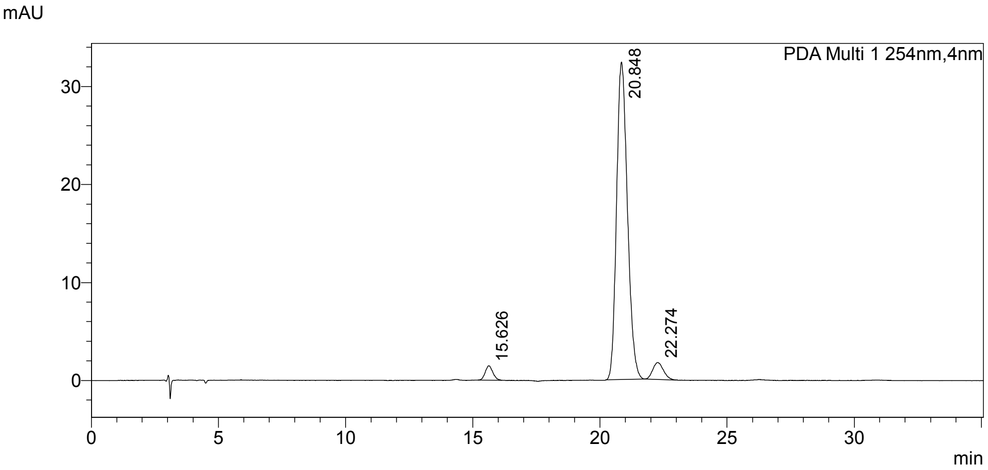

<Peak Table>

PDA Ch1 254nm

Peak#	Ret. Time	Area	Height	Conc.	Unit	Mark	Name
1	15.626	30400	1463	2.914			
2	20.848	962433	32384	92.260			
3	22.274	50344	1723	4.826			
Total		1043177	35571				

^{1}H NMR (600 MHz, $CDCl_3$) and ^{13}C{^{1}H} NMR (126 MHz, $CDCl_3$) spectrum of allyl (2R,9R,E)-11-azido-9-((tert-butyldimethylsilyl)oxy)-2-(3-chloropropyl)undec-4-enoate

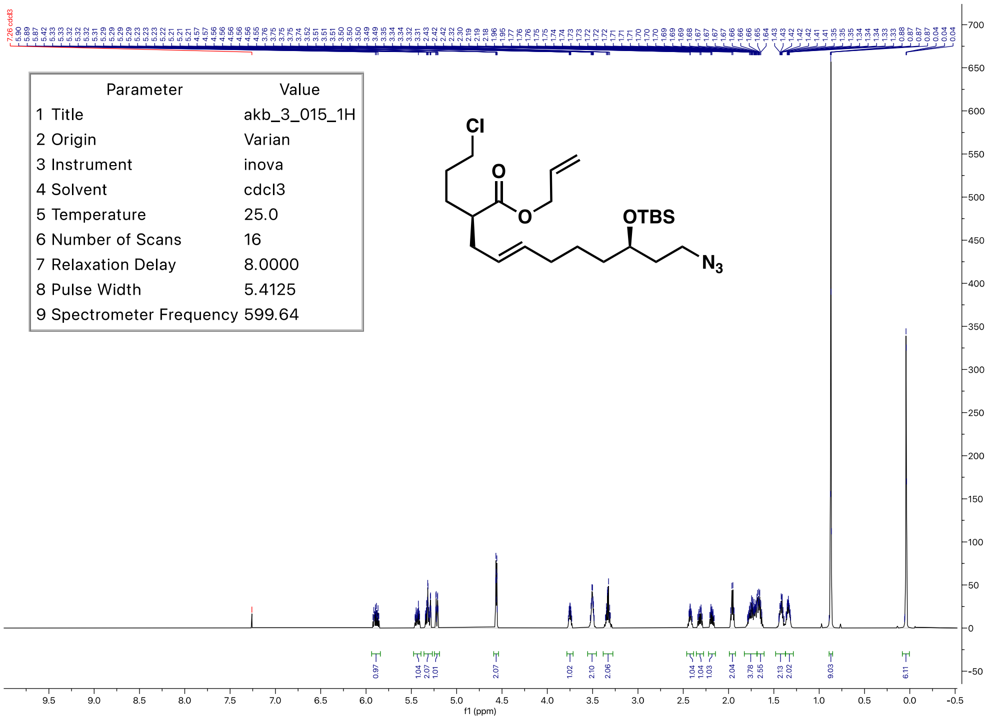

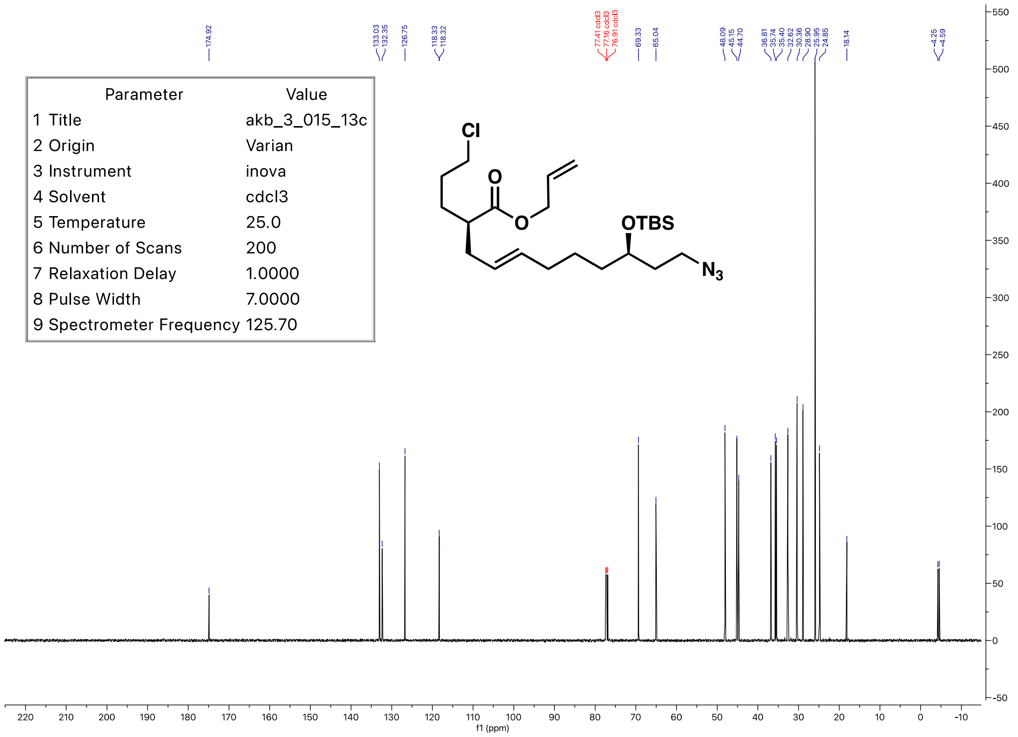

Parameter	Value
1 Title	akb_3_015_13c
2 Origin	Varian
3 Instrument	inova
4 Solvent	cdcl3
5 Temperature	25.0
6 Number of Scans	200
7 Relaxation Delay	1.0000
8 Pulse Width	7.0000
9 Spectrometer Frequency	125.70

^{1}H NMR (600 MHz, $CDCl_3$) and ^{13}C{^{1}H} NMR (126 MHz, $CDCl_3$) spectrum of intermediate 24

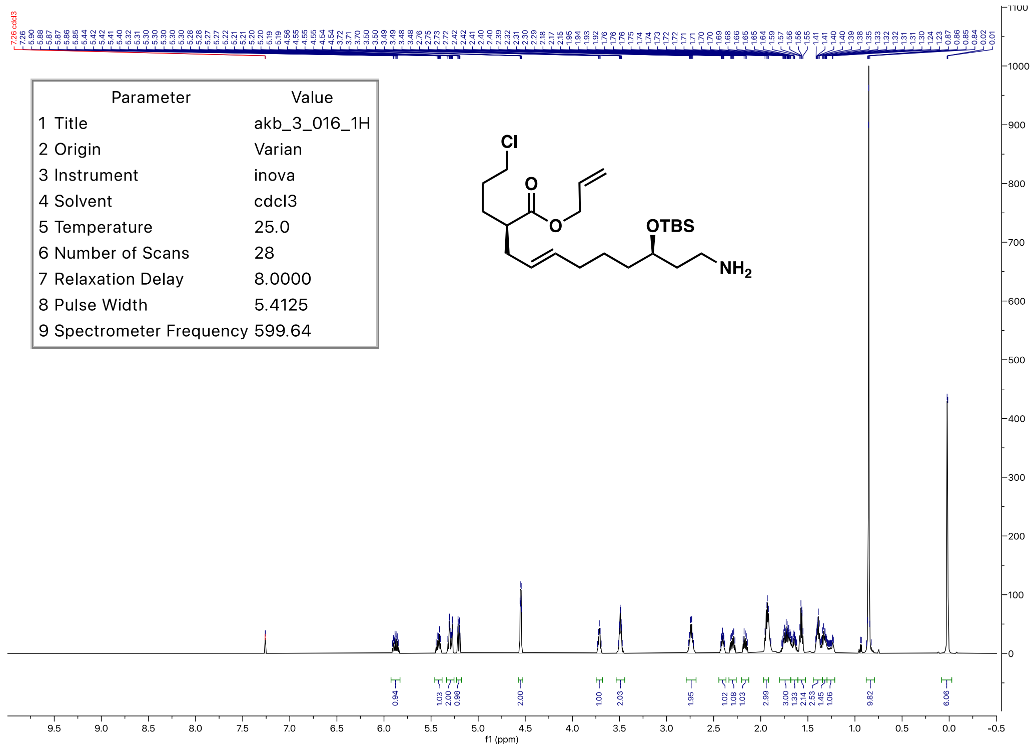

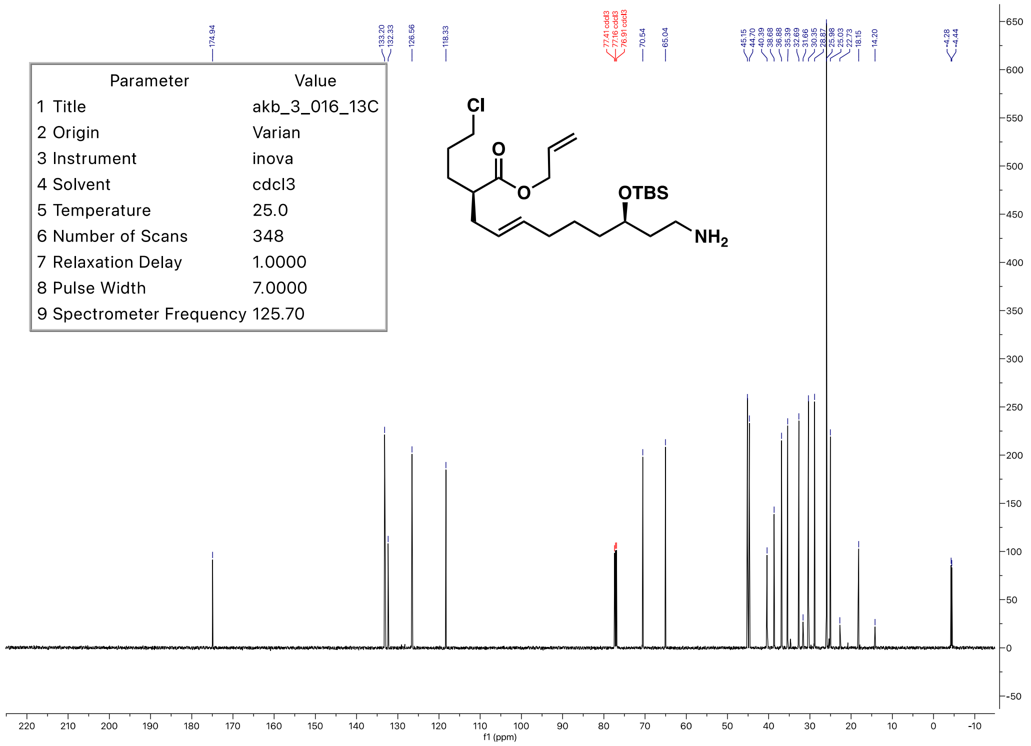

^{1}H NMR (600 MHz, $CDCl_3$) and $^{13}C\{^{1}H\}$ NMR (126 MHz, $CDCl_3$) spectrum of intermediate 25

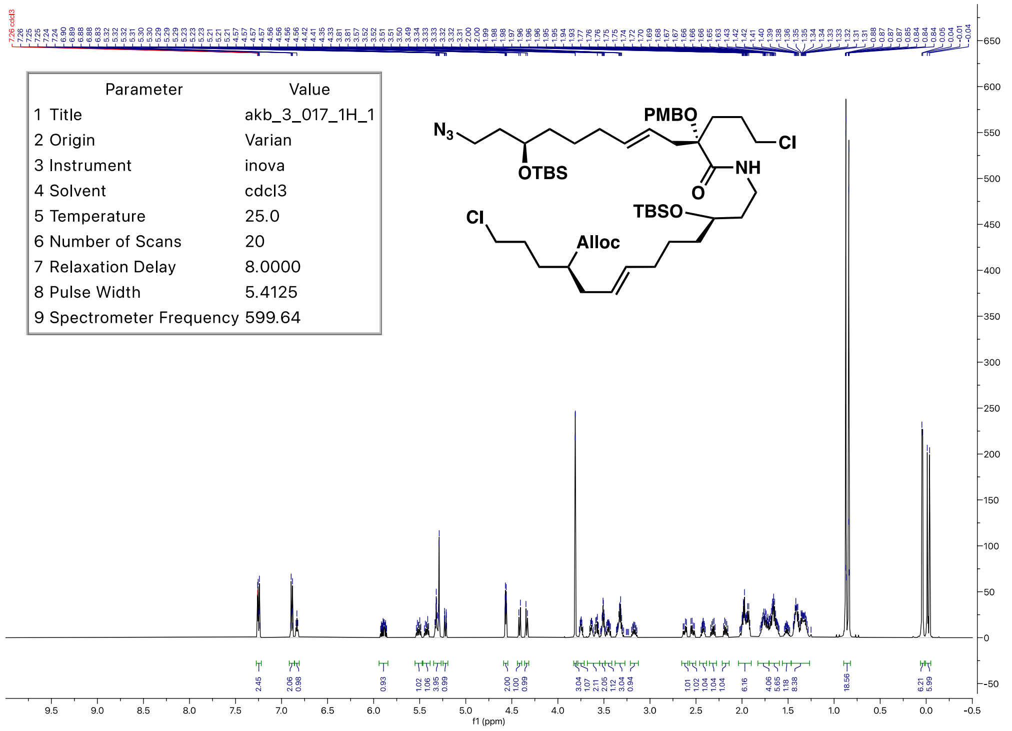

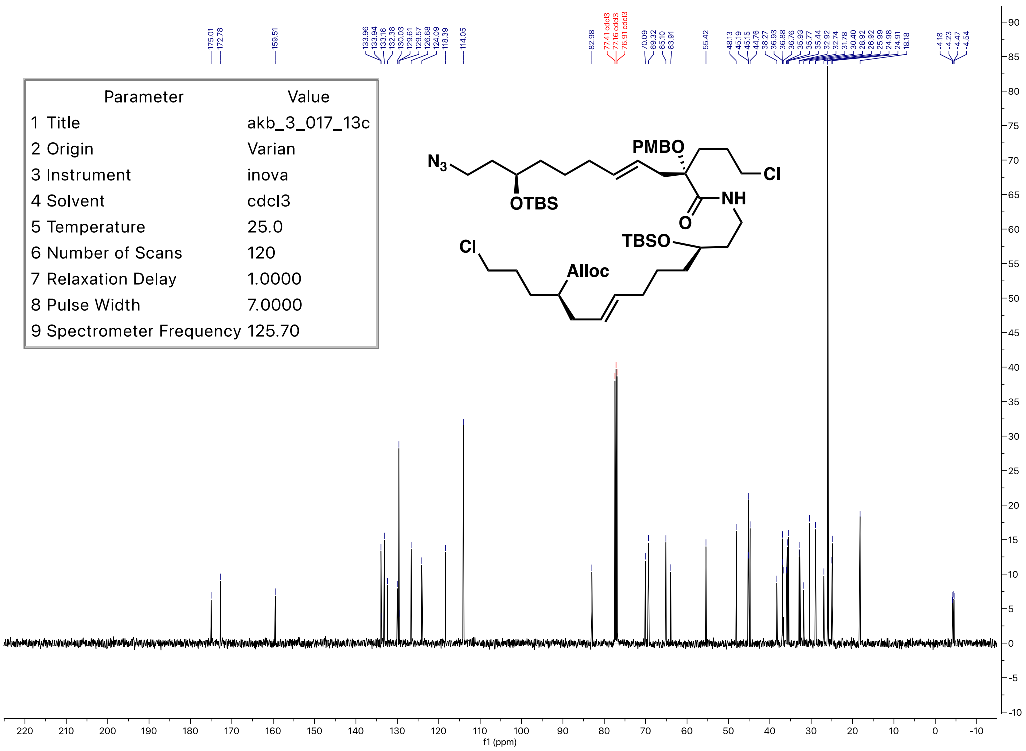
Parameter Value
1 Title akb_3_017_13c
2 Origin Varian
3 Instrument inova
4 Solvent cdcl3
5 Temperature 25.0
6 Number of Scans 120
7 Relaxation Delay 1.0000
8 Pulse Width 7.0000
9 Spectrometer Frequency 125.70
N3
OTBS
PMBO
Cl
NH
O
TBSO
Cl
Alloc
f1 (ppm)

^{1}H NMR (600 MHz, $CDCl_3$) and ^{13}C{^{1}H} NMR (126 MHz, $CDCl_3$) spectrum of intermediate 26

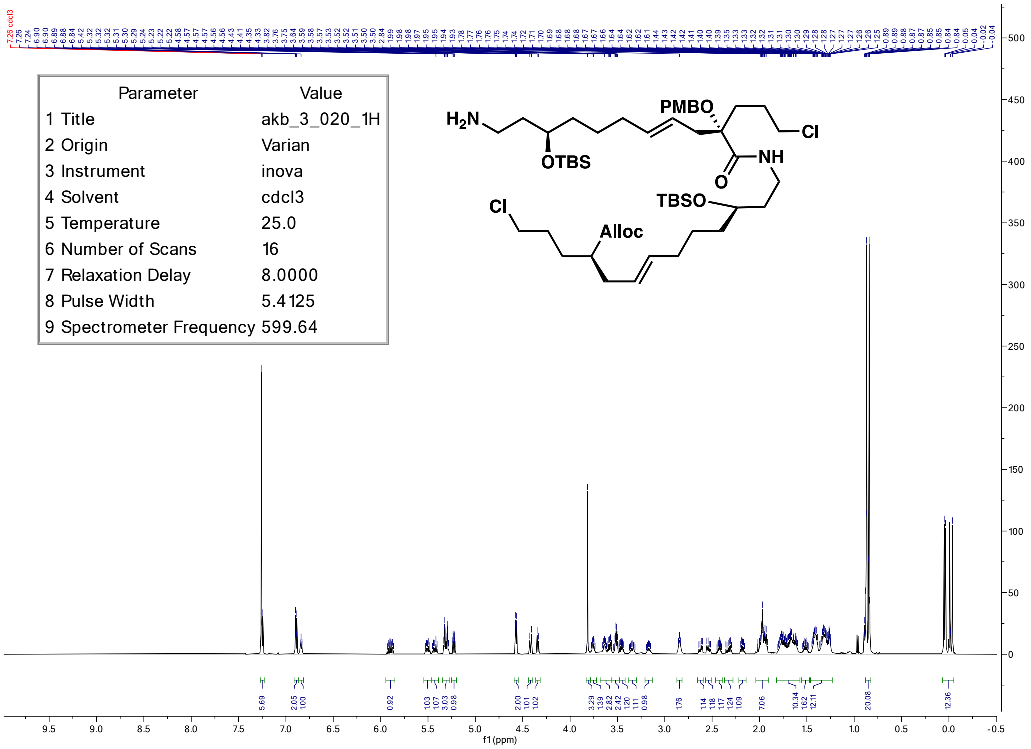

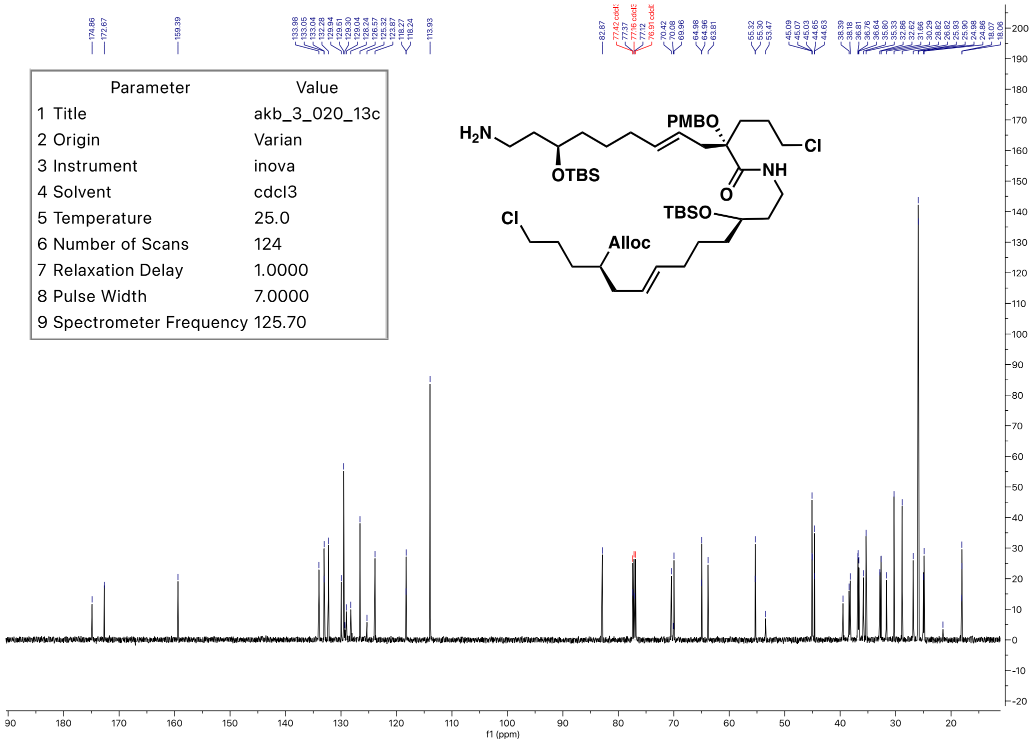

^{1}H NMR (600 MHz, $CDCl_3$) and $^{13}C\{^{1}H\}$ NMR (126 MHz, $CDCl_3$) spectrum of intermediate 27

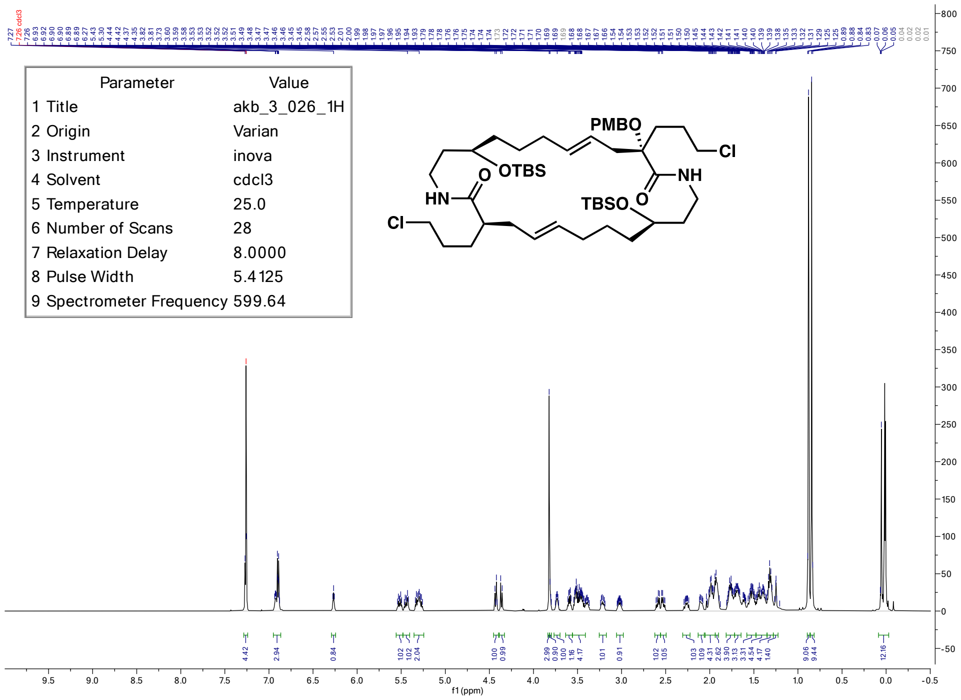

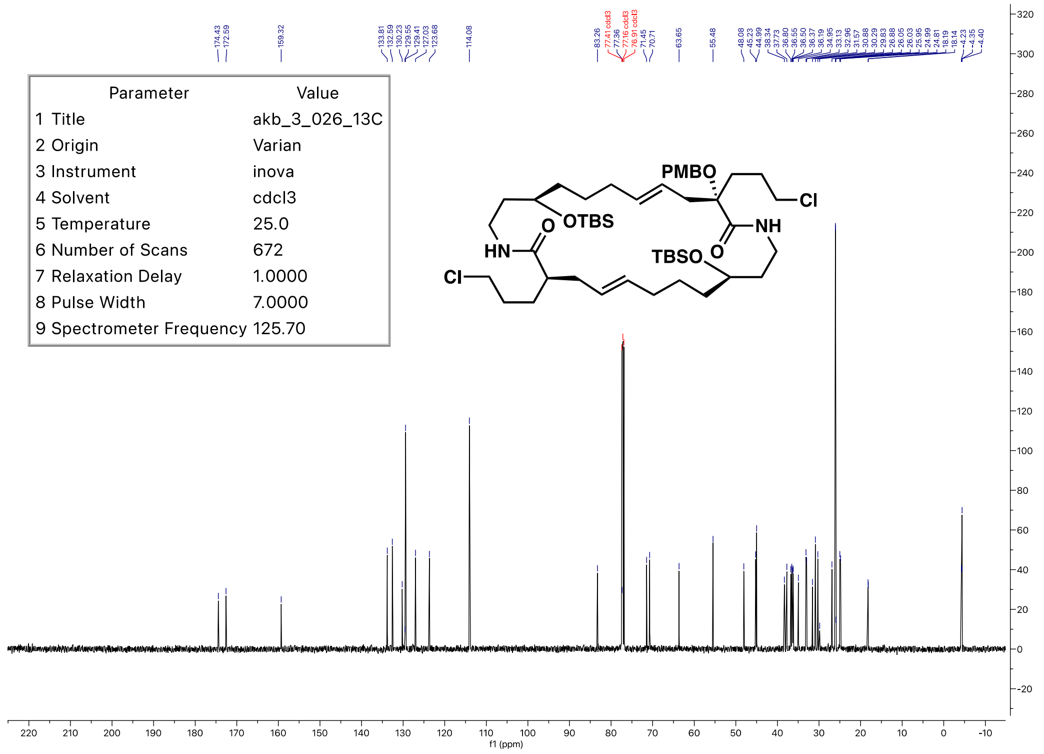

^{1}H NMR (600 MHz, $CDCl_3$) and $^{13}C\{^{1}H\}$ NMR (126 MHz, $CDCl_3$) spectrum of intermediate 28

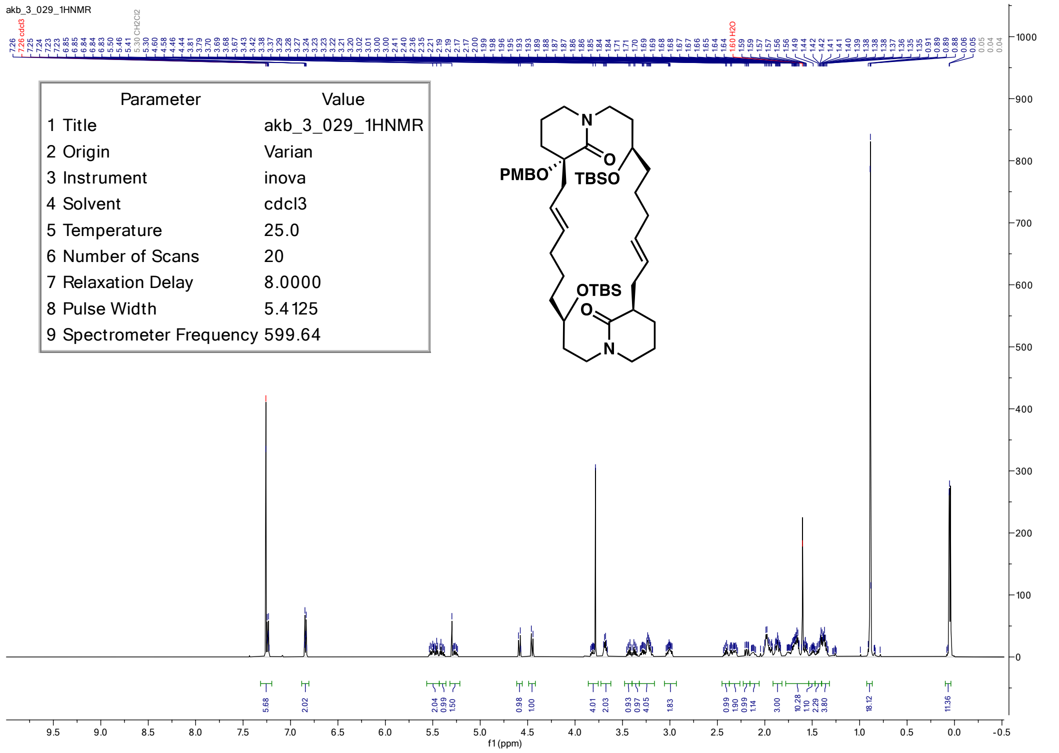

Parameter	Value
1 Title	akb_3_029_1HNMR
2 Origin	Varian
3 Instrument	inova
4 Solvent	cdcl3
5 Temperature	25.0
6 Number of Scans	20
7 Relaxation Delay	8.0000
8 Pulse Width	5.4125
9 Spectrometer Frequency	599.64

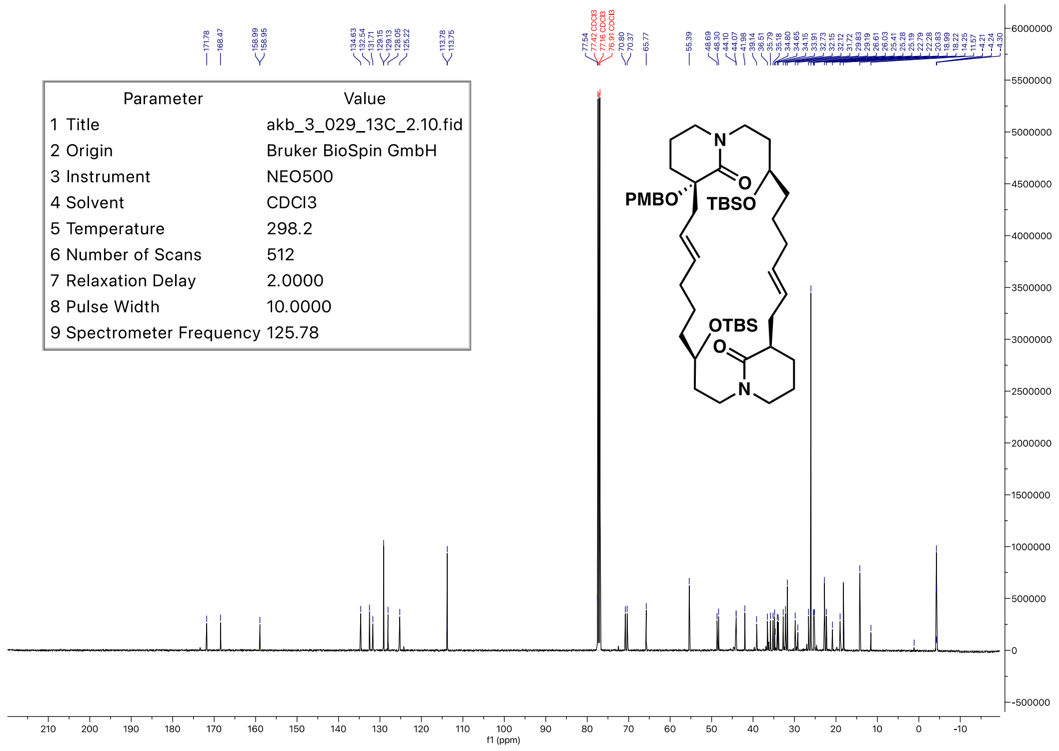

Parameter	Value
1 Title	akb_3_029_13C_2.10.fid
2 Origin	Bruker BioSpin GmbH
3 Instrument	NEO500
4 Solvent	CDCl3
5 Temperature	298.2
6 Number of Scans	512
7 Relaxation Delay	2.0000
8 Pulse Width	10.0000
9 Spectrometer Frequency	125.78

^{1}H NMR (600 MHz, $CDCl_3$) and $^{13}C\{^{1}H\}$ NMR (126 MHz, $CDCl_3$) spectrum of intermediate 29

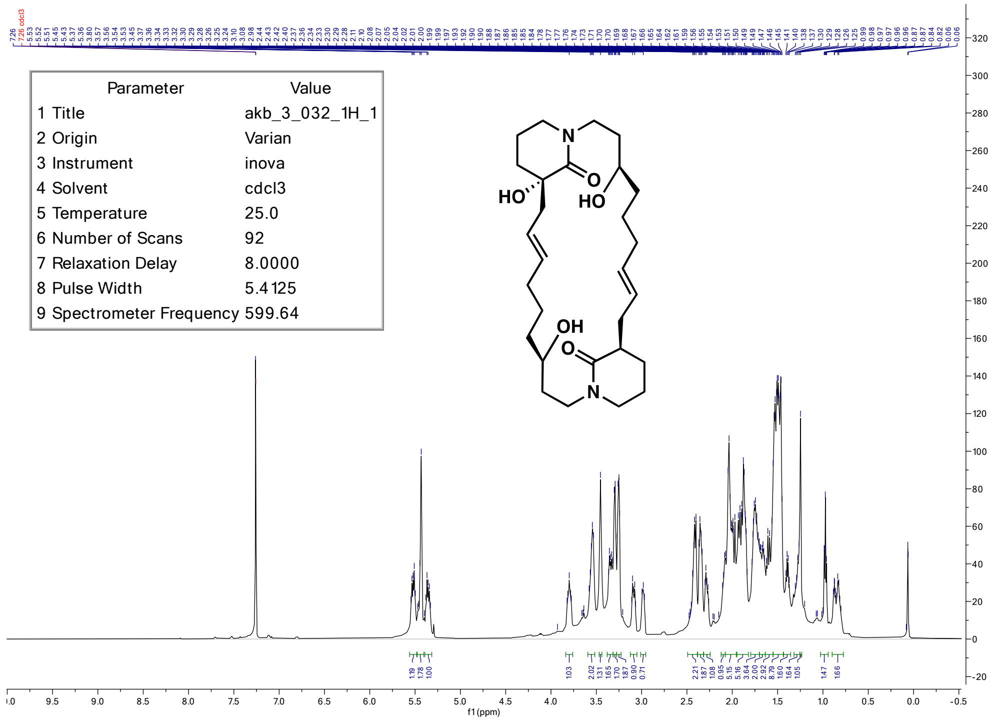

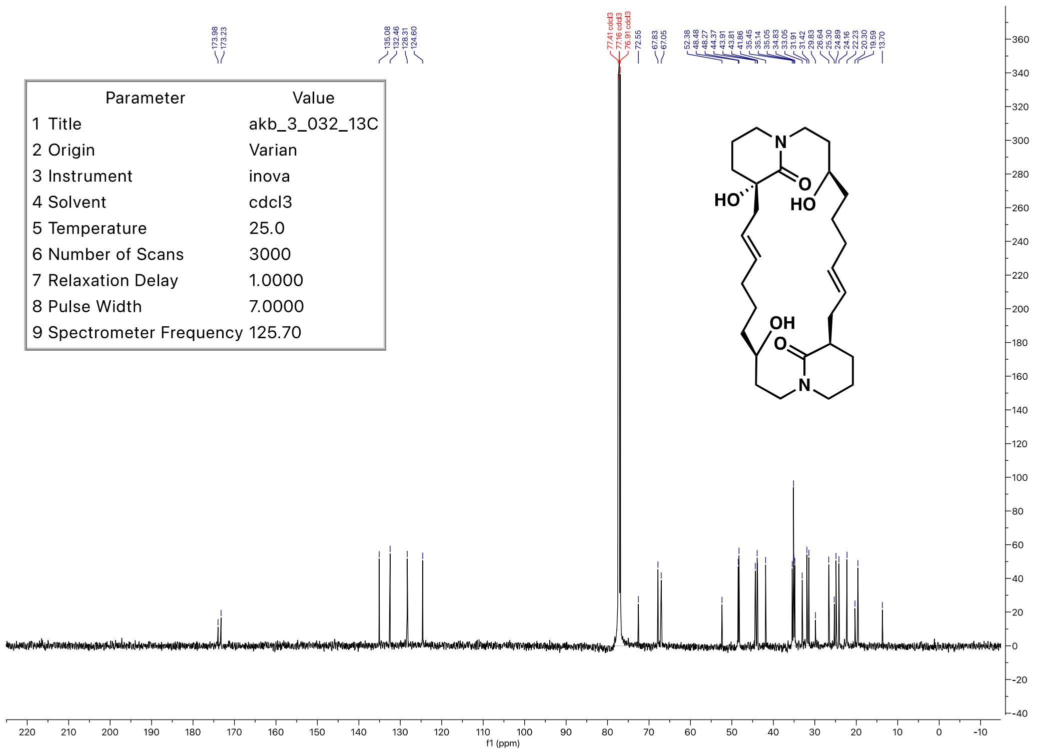
173.98
173.23
135.08
132.46
128.31
124.60
77.41 cdcl3
77.16 cdcl3
76.91 cdcl3
72.55
67.83
67.05
52.38
48.48
48.27
44.37
43.91
43.81
41.86
35.45
35.14
35.05
34.83
33.65
31.91
31.42
29.83
26.84
25.30
24.89
24.16
22.23
20.30
19.59
13.70
Parameter Value
1 Title akb_3_032_13C
2 Origin Varian
3 Instrument inova
4 Solvent cdcl3
5 Temperature 25.0
6 Number of Scans 3000
7 Relaxation Delay 1.0000
8 Pulse Width 7.0000
9 Spectrometer Frequency 125.70
N
O
HO
HO
OH
O
N
f1 (ppm)
